# Label-Free Single-Molecule Pulldown for the Detection of Released Cellular Protein Complexes

**DOI:** 10.1021/acscentsci.2c00602

**Published:** 2022-08-18

**Authors:** Guangzhong Ma, Pengfei Zhang, Xinyu Zhou, Zijian Wan, Shaopeng Wang

**Affiliations:** † Biodesign Center for Biosensors and Bioelectronics, Arizona State University, Tempe, Arizona 85287, United States; ‡ School of Biological and Health Systems Engineering, Arizona State University, Tempe, Arizona 85287, United States; § School of Electrical, Computer and Energy Engineering, Arizona State University, Tempe, Arizona 85287, United States

## Abstract

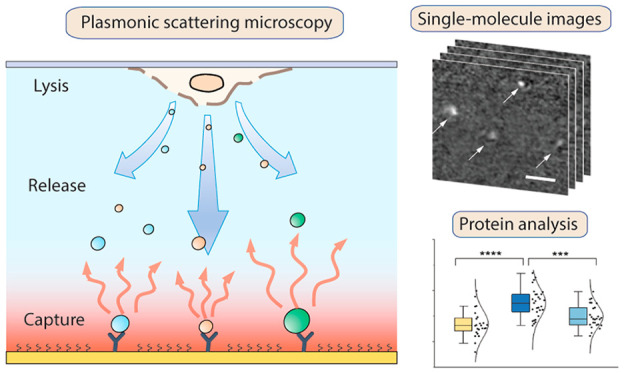

Precise and sensitive detection of intracellular proteins and complexes is key to the understanding of signaling pathways and cell functions. Here, we present a label-free single-molecule pulldown (LFSMP) technique for the imaging of released cellular protein and protein complexes with single-molecule sensitivity and low sample consumption down to a few cells per mm^2^. LFSMP is based on plasmonic scattering imaging and thus can directly image the surface-captured molecules without labels and quantify the binding kinetics. In this paper, we demonstrate the detection principle for LFSMP, study the phosphorylation of protein complexes involved in a signaling pathway, and investigate how kinetic analysis can be used to improve the pulldown specificity. We wish our technique can contribute to uncovering the molecular mechanisms in cells with single-molecule resolution.

## Abstract 2

A plasmonic imaging technique for quantifying intracellular protein complexes at the single-molecule level using only dozens of cells is demonstrated.

## Introduction

In cells, proteins transduce signals and carry out their functions via phosphorylation and assembly into protein complexes.^1−4^ Hence, measuring the abundance and composition of intracellular proteins involved in the pathway has been the most direct way to study these processes and the underlying molecular mechanisms. This task is often accomplished by polyacrylamide gel electrophoresis (PAGE) and Western blot, which separate the proteins and probe the protein of interest using antibodies.^5−8^ Nevertheless, conventional PAGE and Western blot require a substantial number of cells (at least thousands)^9,10^ to reach a sufficient signal-to-noise ratio, which limits the detection of rare cells or low-abundance proteins. Recently, a technique called single-cell Western blot (scWB) has been developed to probe proteins with single-cell resolution.^11,12^ Although scWB has improved detection sensitivity, it denatures protein complexes (like the traditional SDS-PAGE), making it difficult to interpret their native composition and function in cells.

To address these problems, single-molecule fluorescence techniques,^13^ such as single-molecule pulldown (SiMPull),^14−16^ single-molecule coimmunoprecipitation,^17^ and single-molecule fluorescence resonance energy transfer (FRET),^18^ have emerged as sensitive and nondestructive tools for measuring intracellular protein complexes and their composition. However, apart from being time-consuming, the genetically encoded or chemically attached fluorescent tags may introduce a complication by interacting with off-target proteins or altering the interaction affinity.^19,20^ In addition, fluorescence is not applicable for long-term and continuous imaging due to photobleaching, which affects measuring the binding kinetics of proteins.^21^

Here, we present a label-free imaging approach to measure intracellular proteins and protein complexes. The principle resembles SiMPull, except that the proteins are imaged without labels, so we call it label-free single-molecule pulldown (LFSMP). In LFSMP, the molecules released from cells are imaged using plasmonic scattering microscopy (PSM),^22,23^ a single-molecule platform that measures the scattering light from each individual molecule. The scattered light intensity is proportional to the molecular weight, with a dynamic range from ∼100 kDa to several MDa, making LFSMP particularly suitable for protein complex detection. The label-free feature also enables kinetics analysis of the captured complexes. Compared to Western blot technologies, LFSMP measurements can be performed using as few as several cells. And most importantly, LFSMP does not denature the native structure of protein complexes; thus, the composition and function of the complexes are retained.

## Results

### Imaging Single Molecules from Lysed Cells

**Figure 1:**
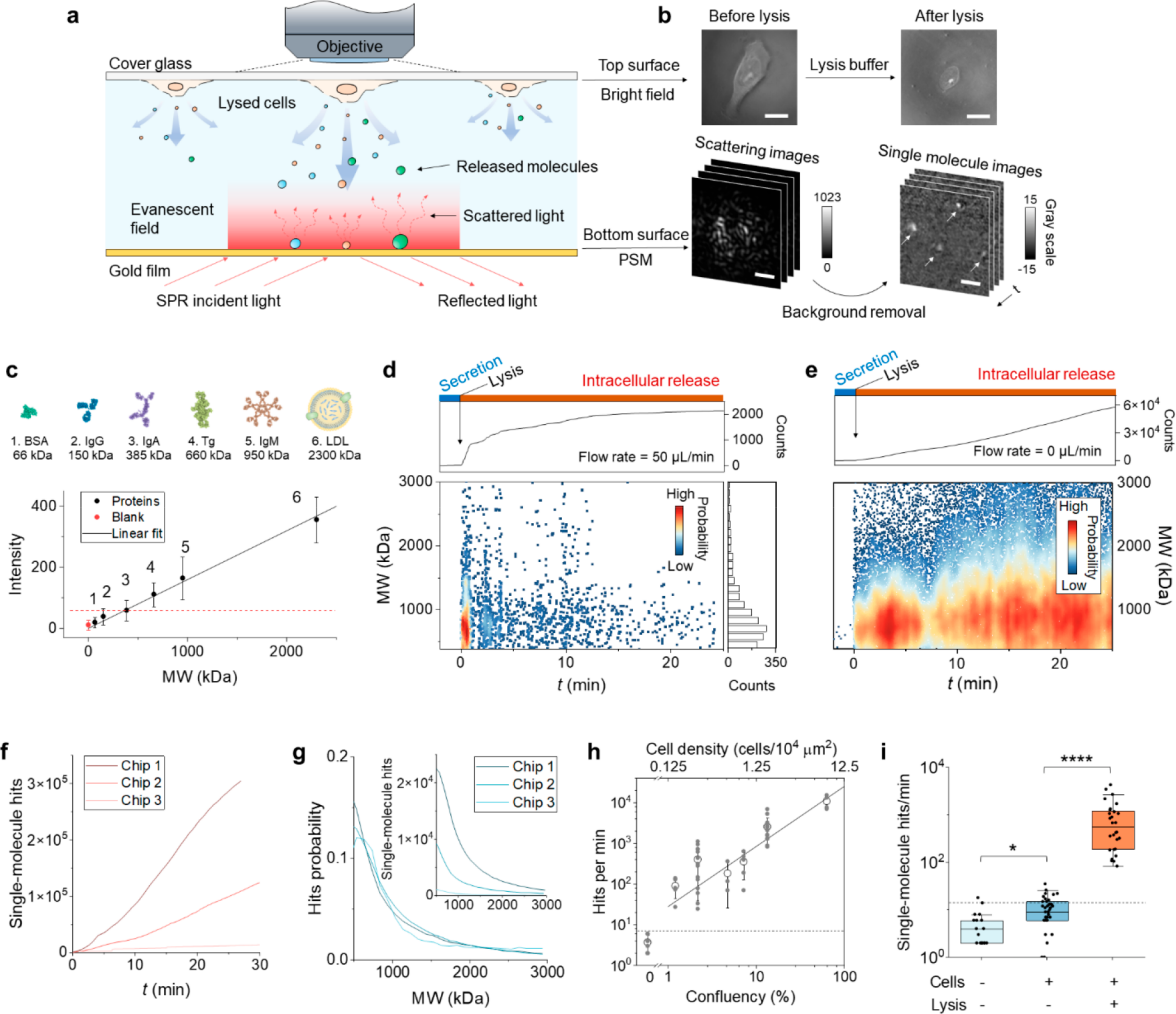
Principle of single-protein imaging and cell lysis detection. (a) Experimental setup. Cells are cultured on the top glass surface of the flow chamber. After introducing lysis buffer, intracellular molecules are released and diffuse to the bottom gold film surface. An incident light is guided to the gold film by a prism to excite SPR on the surface. Single molecules hit the surface and scatter the light in the evanescent field, which is imaged by a CMOS camera via an objective above the chamber. (b) The top surface imaged in a bright field showing the morphology of a single cell before and after lysis. Scale bar, 15 μm. The bottom surface recorded in PSM mode shows the scattering of the surface. Single molecules are revealed after removing the background by differential imaging, where each bright spot indicated by the arrows is a single protein or complex molecule. Scale bar, 3 μm. (c) The conversion from scattered light intensity to molecular weight (MW) is calibrated using protein samples in PBS solution. Error bars represent the mean ± standard deviation (s.d.) obtained from >1000 single molecules. The blank sample is pure PBS solution, showing the system noise level. The red dashed line marks the detection limit of the PSM imaging technique, defined by mean + 3 × s.d. of the blank. (d) Temporal profile of the release of intracellular molecules during cell lysis. Lysis buffer was injected at *t* = 0 and flowed at 50 μL/min in the chamber. Top panel: cumulative single-molecule hits on the surface. Bottom left panel: mass distribution of single molecules over time after the lysis, where each point is from a single molecule. Bottom right panel: MW histogram obtained by projecting the single-molecule data. (e) Temporal profile of single-molecule hits without flow after cell lysis. The flow was stopped immediately after introducing lysis buffer. The cell confluences in (d) and (e) are 10 and 15%, respectively. (f) Cumulative single-molecule hits obtained from three sensor chips with random cell confluence. (g) Normalized mass distribution of released single molecules obtained from the three chips. The inset shows the original distribution. (h) Hitting rate as a function of confluency or cell density. The data are plotted in log–log scale and fitted linearly (solid line). Data within the same group were measured with one chip but using different regions of interest (ROIs). The 0 confluence was measured using a chip without cells. Error bars represent mean ± s.d. The dashed line marks mean + 2 × s.d. of the 0 confluency. (i) Control experiments using chips without cells or lysis buffer. From left to right: *n* = 17 (on 3 chips), 37 (on 7 chips), and 28 (on 8 chips). The dashed line shows mean + 2 × s.d. of the cell-free group. *P < 0.05; ****P < 0.0001.

The stability of intracellular proteins and complexes is known to decrease when extracted to extracellular evironments.^24,25^ To collect the released molecules rapidly and efficiently after cell lysis, we fabricated a thin flow channel (51 μm in height) with adherent live cells cultured on the top glass surface (Figure 1a). After introducing lysis buffer, the cells are lysed *in situ*, and the intracellular molecules, including proteins and protein complexes, are released into the channel and diffuse to the bottom gold film surface. We use PSM to image the released single molecules at the bottom. The configuration of the setup is shown in Figure S1.^23^ Briefly, an incident laser is coupled to the gold surface via a prism, which excites surface plasmon resonance (SPR) and the associated evanescent field on the surface. The scattered light by the molecules as well as by the surface roughness within the field are collected by an objective on top of the flow channel and imaged by a CMOS camera (Figure 1b, bottom panels). Single-molecule images are obtained after background removal (see below). The top surface of the channel can be imaged using the same objective under a bright field to locate the cell and observe the morphology change before and after lysis (Figure 1b, top panels). The evanescent field at the bottom is confined to the surface within ∼100 nm; thus, the cells and molecules in the bulk solution could not be imaged by PSM.

The capability of PSM for imaging single molecules has been demonstrated previously.^22,23^ Here, we first measured several pure protein samples with molecular weight (MW) ranging from 66 kDa to 2.3 MDa to establish a relationship between MW and scattering intensity. The single molecules landing on the surface generated bright spots (Figure 1b, bottom panels) and the image intensity of the spots were measured. Figure 1c shows a linear relationship between the MW and image intensity of the proteins, which is expected according to the PSM imaging principle.^22^ Among the six measured proteins, immunoglobulin G (IgG), immunoglobulin A (IgA), thyroglobulin (Tg), immunoglobulin M (IgM), and low-density lipoprotein (LDL) could be readily imaged with single-molecule resolution (Figure S2). However, bovine serum albumin (BSA) did not have enough signal-to-noise ratio (SNR) due to its small size and insufficient scattered photons. A blank sample containing only phosphate-buffered saline (PBS, the solvent of the protein samples) was used to determine the system noise level. The mean + 3 × standard deviation (s.d.) of the blank was defined as the detection limit, which was 385 kDa in MW. We use this number as a threshold to exclude small proteins or low-SNR spots for the following protein complex measurements.

We next studied the cell lysis process with PSM. The cells were cultured on the top surface at a confluence of 10% or 1.25 cells/10^4^ μm^2^, and the bottom surface was blocked with BSA. Live cell imaging buffer was flowed in the channel at a constant flow rate of 50 μL/min to maintain cell viability. Before cell lysis, only a few secreted molecules were imaged on the bottom surface with a hitting rate of <1 hit/min, which was due to the low level of macromolecule secretion. In contrast, upon introducing cell lysis buffer, a sudden increase of released molecules was observed with ∼1000 hits/min, indicating immediate lysis of the cells. Some extremely strong scattering signals could be occasionally observed, which was likely due to cell debris and organelles (Supplementary Movie 1) because the detergent we used was mild.^14^ The hit rate is proportional to the protein concentration, as confirmed by a calibration measurement (Figure S3), and 1000 hits/min is equivalent to 550 nM according to the calibration. Figure 1d (top panel) shows the total number of single-molecule hits before and after the lysis. The release was most active in the first 1 min and then gradually declined over the next 25 min due to the depletion of intracellular molecules (Supplementary Movie 2). The MW of each single molecule is determined using the curve in Figure 1c, and a release mass profile is shown in Figure 1d (bottom panels). Note that the mass profile only shows molecules with MW > 385 kDa, because smaller molecules with insufficient SNR are discarded. Another observation is that larger molecules (e.g., >1500 kDa) are more likely to release in the first 10 min. After 15 min, over 95% of the molecules are smaller than 1000 kDa. This might be caused by the disassembly of protein complexes after the breakdown of the intracellular environment.^24^

Because most molecules are flushed away quickly by the flow after the lysis, their in-channel retention time is limited, which reduces the pulldown probability. This is particularly undesirable when measuring low-abundance proteins and complexes. To solve this problem, we stopped the flow once the channel was filled with lysis buffer, such that most of the released molecules could be trapped inside the channel and could have more interactions with the surface. We repeated the lysis experiment and measured the number of released molecules over time as we did in Figure 1d but without the flow (cells at 15% confluence), and the result is shown in Figure 1e. Although the lysis was finished in 1 min, the released molecules kept hitting and interacting with the surface with no decline even after 25 min (Supplementary Movie 3). Therefore, we adopted this method for our measurements to increase the reaction efficiency. Another important factor that affects the hitting rate is cell confluence. By measuring three sensor chips with different confluences, we found substantial variations in the hitting rate (Figure 1f). However, the mass distribution of the three measurements shows similar profiles, suggesting that the MW of released molecules is irrelevant to the confluency (Figure 1g). To establish a quantitative relationship between cell confluence and single-molecule hitting rate, we counted the number of molecules at different cell confluences from 1 to 60% (Figure 1h). A control experiment was conducted using a channel without cells with 3.7 ± 1.8 hits/min detected, which was solely due to the impurities in the buffer. The detection limit, defined by mean + 2× s.d., is equivalent to 0.40% confluence or ∼5 cells in a 1 mm^2^ region. We repeated the lysis measurement >25 times and summarized the result in Figure 1i. The plot shows the comparison of hitting rate between lysed cells and controls (unlysed or without cells), where the lysed cells have markedly more molecules released. The unlysed cells were also found to secrete molecules but at a much lower level.

### Specific Detection of Single-Protein Complexes

We studied the mammalian target of rapamycin (mTOR) to demonstrate specific pulldown of single molecules from lysed cells. mTOR is an intracellular protein that plays a central role in regulating metabolism, growth, and proliferation, by forming two structurally and functionally distinct complexes, mTOR complex 1 (mTORC1) and mTOR complex 2 (mTORC2). Malfunction of mTORC has been linked to diseases such as cancer and diabetes.^26,27^ Both mTORC1 and mTORC2 are multimeric complexes with molecular weights estimated to be ∼1000 and 1500–2000 kDa, respectively.^16,28,29^

**Figure 2:**
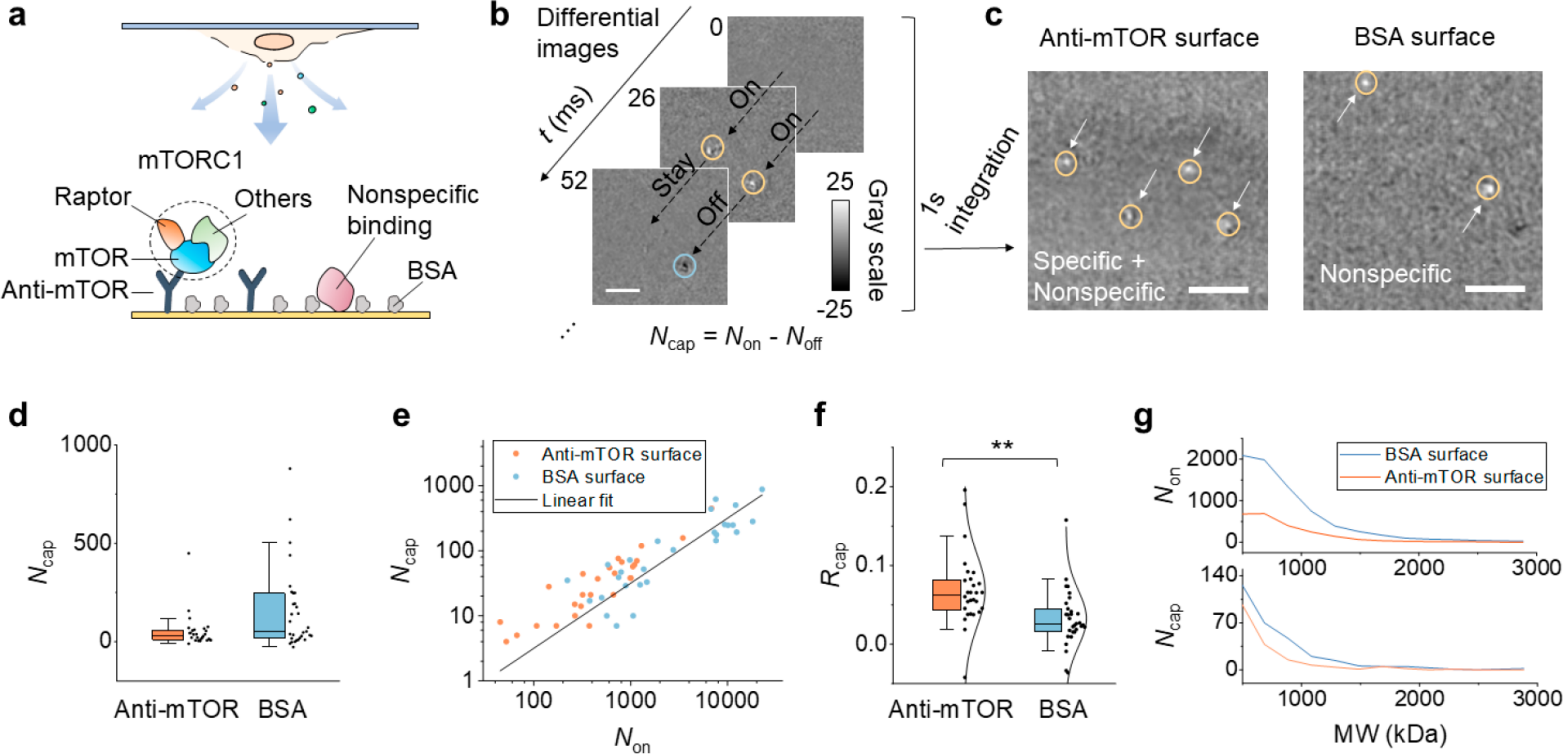
Specific detection of released single mTORC1. (a) mTORC1, an intracellular protein complex consisting of mTOR, Raptor, and other components is specifically captured to the anti-mTOR functionalized surface. Although the surface is blocked with BSA, some large molecules can still bind to the surface nonspecifically and be imaged by PSM. (b) Representative differential images showing the dynamic binding and unbinding of single-protein complexes. Scale bar, 3 μm. The bright spot and the dark spot in the image indicate the molecule hitting or leaving the surface, respectively. The total number of captured molecules (*N*_cap_) in a measurement is defined by *N*_cap_ = *N*_on_ – *N*_off_. (c) Integration of the differential images for 1 s. The arrows mark the position of the captured molecules. The left and right panels show the result of using an anti-mTOR surface and BSA surface, respectively. Scale bar, 3 μm. (d) The numbers of captured molecules on an anti-mTOR surface and BSA surface. Each data point is obtained from an individual measurement; *n* = 28 (on 9 chips) and 31 (on 10 chips) for the anti-mTOR and BSA groups, respectively. For each measurement, the cell confluence is random, and the detection time ranges from 30 s to 2 min. (e) The positive correlation between *N*_cap_ and *N*_on_. (f) Capture ratio (*R*_cap_ = *N*_cap_/*N*_on_) is used to describe the binding ability, with anti-mTOR showing a significantly higher ratio than BSA. **P < 0.01. The data are fitted with a normal distribution (solid curves). (g) Representative mass distribution curves of released molecules (top) and captured molecules (bottom) for anti-mTOR and BSA surfaces.

To capture mTORC, we functionalized the channel bottom surface with anti-mTOR and blocked the nonspecific sites with BSA, which is a common blocker in immunoassays (Figure 2a). We determined the captured molecules by analyzing the binding and unbinding events. To be specific, an image sequence was recorded after cell lysis, and a differential image sequence was obtained by subtracting the previous frame from each frame. Common backgrounds were removed in the differential images so that the single molecules were revealed. An example is shown in Figure 2b. Each bright spot indicates a single molecule hitting on the surface, while each dark spot is caused by the molecule leaving. By counting the total number of hitting (*N*_on_) or leaving (*N*_off_) events over a period of time, the net counts of captured molecules is obtained by *N*_cap_ = *N*_on_ – *N*_off_. To check whether the captured molecules were caused by specific binding, we compared the *N*_cap_ of the anti-mTOR coated surface and that of a BSA coated surface. Molecules bound to BSA should be nonspecific; thus, the BSA surface serves as a negative control. As an illustration, Figure 2c shows integration of the differential images over 1 s for the anti-mTOR surface and the BSA surface. Both surfaces have captured molecules, suggesting strong nonspecific binding of released proteins/complexes to BSA. We obtained *N*_cap_ from >25 measurements and found anti-mTOR did not show a higher *N*_cap_ statistically (Figure 2d). This result does not mean we could not distinguish the specific binding and nonspecific binding. As discussed above, the total number of released molecules (or hits) is different for each measurement, which stems from variations in cell confluence, detection time, and lysis efficiency. In fact, *N*_cap_ is positively correlated with the total hits (*N*_on_) (Figure 2e). As such, the real binding level should be *N*_cap_ normalized by *N*_on_. Figure 2f shows a normalized *N*_cap_ for anti-mTOR and BSA surfaces, where *R*_cap_ = *N*_cap_/*N*_on_. The data follows a Gaussian distribution after normalization, and anti-mTOR is significantly higher than BSA with *P* = 0.0015. The specific and nonspecific binding of mTORC can only be distinguished statistically because the cell lysate contains many kinds of proteins and complexes, which are impossible to be blocked completely. This high nonspecific background significantly reduces the resolving power, making it difficult to differentiate specific/nonspecific binding in a single chip measurement. We also studied the mass distribution of the released molecules (Figure 2g). The MW profiles determined using all the released molecules are similar for an anti-mTOR surface and BSA surface, which indicates surface modification does not affect MW detection. However, by plotting the MW distribution of the captured molecules, we did not find clear bands for mTORC at the predicted MW because of the dominant nonspecific binding. It is also possible that some components in mTORC are partially dissociated in the lysis buffer (Supplementary Note 1 and Figure S4). The specific capture of mTORC was also confirmed with a fluorescence measurement (Supplementary Note 2 and Figure S5). Taken together, the above results suggest our method can pull down cellular molecules with good specificity.

### Counting Single-Protein Complexes for a Signaling Pathway Study

**Figure 3:**
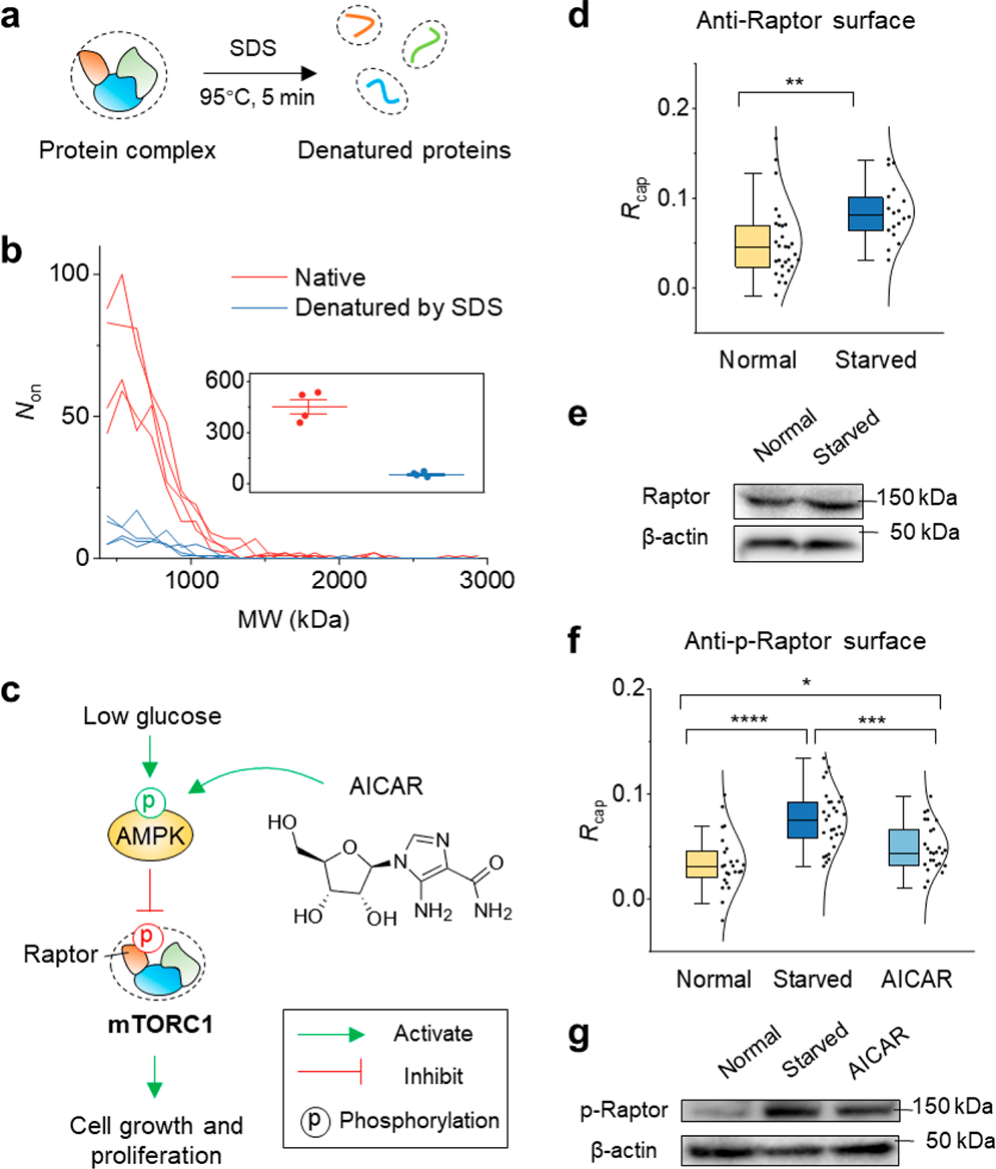
Study of the AMPK/mTORC1 signaling pathway by counting single-protein complexes. (a) Schematic showing the protein complex in cell lysate is denatured into peptides using a common SDS-PAGE preparation protocol. (b) The mass profile of protein complexes detected in native cell lysate and denatured cell lysate. Both lysates were diluted 100 times with PBS and measured for 30 s. Four measurements were performed using different sensor chips. The inset shows the total number of detected molecules. The error bar represents mean ± s.d. (c) A brief schematic of the AMPK regulated mTORC1 signaling pathway. (d) Normal cells and starved cells were lysed on top of an anti-Raptor coated surface. *R*_cap_ was determined from *n* = 29 (on 3 chips) and *n* = 18 (on 3 chips) measurements for normal cells and starved cells, respectively. (e) Western blot result showing the Raptor level in normal cells and starved cells. (f) Normal cells, starved cells, and AICAR treated cells were lysed on top of an anti-phospho-Raptor antibody functionalized surface, with *n* = 24 (on 8 chips), 29 (on 4 chips), and 26 (on 5 chips) measurements, respectively. The *R*_cap_ values were compared. (g) Western blot result showing the phospho-Raptor level in normal, starved, and AICAR treated cells. *P < 0.05; **P < 0.01; ***P < 0.001; ****P < 0.0001.

Protein complexes are involved in many signaling pathways. Probing such protein complexes usually requires denaturation, separation, and detection by SDS-PAGE and Western blot, which totally breaks the native structure of the complexes (Figure 3a). LFSMP provides a direct avenue to measure the protein complexes with minimal perturbation to the native structures and with single-molecule sensitivity at the same time. To study the effect of denaturation on the size of protein complexes, we harvested cells from a culture flask (∼1 million cells), lysed the cells and collected the lysate, and denatured a fraction of the lysate by incubating with SDS at 95 °C for 5 min. After dilution 100× with PBS, the sample was immediately loaded into the channel and imaged with PSM. Native lysate (also 100× diluted with PBS) without SDS treatment was measured as well (Supplementary Movie 4). The counts and mass distribution are shown in Figure 3b. In the native lysates, 10 times more counts were found, which suggests over 90% of protein complexes were denatured into smaller pieces after SDS treatment.

Next, we studied protein phosphorylation in the AMPK/mTORC1 pathway by pulling down the mTOR complexes. The AMPK/mTORC1 pathway is responsible for sensing energy and nutrients and regulating cell growth and proliferation (Figure 3c).^27,30^ Under low-glucose conditions, AMPK is activated via phosphorylation, which reduces the activity of mTORC1 by phosphorylating one of its components, Raptor. We used an anti-Raptor coated chip to capture the released mTORC1 from normal cells and glucose-starved cells. Anti-Raptor can bind to both phosphorylated and unphosphorylated Raptor, thus reflecting the total Raptor level. A markedly higher level of Raptor (*P* = 0.0042) was found for the starved group (Figure 3d). We also performed the same measurement using cell lysate with conventional SDS-PAGE and Western blot, but little difference was found between the normal cells and starved cells (Figure 3e). The Western blot result agrees with previous findings that the total abundance of Raptor should not be altered by glucose deprivation.^31^ However, the same report also reveals that the amount of mTOR associated Raptor is decreased in glucose-free medium, which is contrary to our LFSMP result. This discrepancy may indicate that the protein complex we detected is not Raptor associated mTORC1 but another complex that contains Raptor. In other words, we infer Raptor may detach mTORC1 and bind to other proteins under glucose-deprived conditions. Further investigations should made to test our assumption.

The deprivation of glucose also phosphorylates Raptor on Ser-792 in mTORC1. To investigate this process, we used phospho-Raptor antibody (anti-p-Raptor) for the pulldown measurement. A higher level of phosphorylated Raptor (p-Raptor) was observed in starved cells compared with normal cells (*P* = 2.2 × 10^–6^) (Figure 3f), consistent with the literature.^31^ We also performed a positive control by using 5-aminoimidazole-4-carboxamide (AICAR) to stimulate AMPK phosphorylation.^32^ As expected, the p-Raptor level was elevated compared with the normal cells (*P* = 0.041) but still much lower than the starved group (*P* = 4.2 × 10^–4^). The results were confirmed by Western blot using cell lysates (Figure 3g). Together, our findings suggest that LFSMP can measure the native format of protein complexes and be combined with traditional Western blot assays to decipher the role of protein complex in signaling pathways.

### Differentiate Specific and Nonspecific Binding by Analyzing the Binding Kinetics

**Figure 4:**
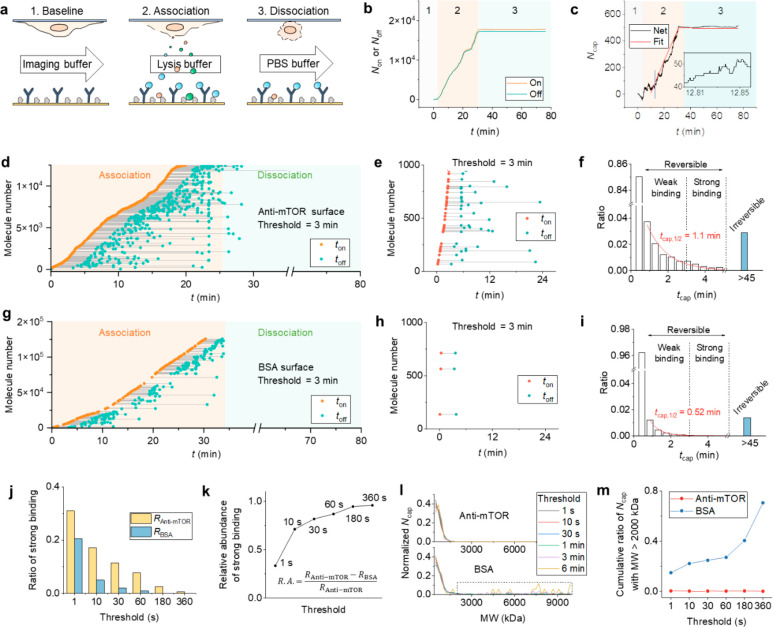
Real-time detection of mTORC1 binding to anti-mTOR. (a) Detection protocol. The number of released complexes is continuously counted before (baseline), during (association), and after (dissociation) cell lysis. The arrow and the box indicate flow at 50 μL/min and no flow, respectively. (b) The numbers of complexes binding to (*N*_on_) or unbinding from (*N*_off_) the anti-mTOR functionalized surface during the three processes. The complex concentration is calculated to be 424 nM. (c) The numbers of complexes captured on the anti-mTOR surface (*N*_cap_). The data are fitted to the first-order kinetics (red curve). The inset is a zoom-in of the section marked by the blue line, showing single-molecule events. (d) Correlating the binding (*t*_on_) and unbinding timestamps (*t*_off_) for individual complexes. The *t*_on_ and *t*_off_ from the same molecule are connected with a line, and the capture time is defined by the length of the line (*t*_cap_ = *t*_on_ – *t*_off_). The plot only shows data with *t*_cap_ > 3 min for clarity. (e) A zoom-in of (d) showing the binding and unbinding of representative single-protein complexes with *t*_cap_ > 3 min. (f) Histogram showing the distribution of *t*_cap_ determined in (d). The molecule number for each bin is normalized by *N*_on_. The histogram is fitted to an exponential decay model (red curve), and the decay constant is 1.1 min. The reversible domain includes complexes that have both *t*_on_ and *t*_off_ identified during the measurement, while the irreversible domine illustrates complexes that only have *t*_on_ and does not unbind within the 45 min dissociation phase. The dashed line is a time threshold at 3 min separating the reversible domain into a weak binding regime and strong binding regime. (g) Control experiment using a BSA coated surface. (h) A zoom-in of (g) showing representative single-protein complexes with *t*_cap_ > 3 min. (i) Histogram showing the distribution of *t*_cap_ determined in (g). The decay constant is fitted to be 0.52 min (red curve). (j) The ratios of strong binding in reversible interactions for anti-mTOR and BSA surfaces at different thresholds. (k) Relative abundances between complexes captured by anti-mTOR and BSA using different thresholds. (l) Mass distributions of captured complexes by anti-mTOR and BSA with different applied thresholds. (m) The ratios of high-MW (>2000 kDa) complexes using different thresholds.

The label-free feature of LFSMP allows us to analyze the single-molecule binding in detail. Like the traditional ensemble SPR technique, we measured the binding between anti-mTOR and released mTORC in three phases, i.e., baseline, association, and dissociation (Figure 4a). In the baseline phase, imaging buffer was flowed in the channel to keep cell viability. Only a few secreted proteins were found on the bottom surface. Then, we initiated the association phase by introducing lysis buffer into the channel. The flow was immediately paused after filling the channel with lysis buffer, and the released molecules were trapped in the channel. Finally, after sufficient single-molecule binding/unbinding events were recorded, PBS buffer was flushed over the surface to wash off the weakly bound molecules. Using the aforementioned counting method, we obtained *N*_on_ and *N*_off_ for the mTORC–anti-mTOR interaction as a function of time (Figure 4b). Their difference, determined by *N*_cap_ = *N*_on_ – *N*_off_, shows the net captured molecules or the binding kinetics curve (Figure 4c). By fitting the curve to the first-order kinetics, the association rate constant (*k*_a_), dissociation rate constant (*k*_d_), and dissociation constant (*K*_D_) are found to be 1.9 × 10^2^ M^–1^ s^–1^, 1.0 × 10^–6^ s^–1^, and 5.2 nM, respectively, which are indicative of strong binding. Note that the beginning of association is mass transfer limited due to cell lysis, but we neglect this effect considering the lysis was rapid (∼1 min) compared with the whole association phase (∼30 min).

The curve in Figure 4c includes both specific and nonspecific binding, which is challenging for traditional ensemble biosensors to discriminate. We achieved this goal by correlating the spatiotemporal coordinates of the binding and unbinding events. For each binding or unbinding event, we extracted the spatial coordinates (*x*, *y*), image intensity (or MW), and the timestamp (*t*) (Figure S7). After comparing the *x*, *y*, MW, and *t* pairwise, we identified the binding event and unbinding event from the same single molecule. The criteria for assigning two events (1 and 2) to the same molecule are (1) the distance between (*x*_1_, *y*_1_) and (*x*_2_, *y*_2_) is smaller than the diffraction limit; (2) the difference of MW should fall within the measurement error; and (3) binding always occurs before unbinding (*t*_1_ < *t*_2_). These analyses are feasible only if the imaging system is stable enough; otherwise, the imaging region would drift away, and the molecule is lost. Our prism based PSM system owns excellent stability with negligible or correctable drift even after 1 h of continuous image recording (Figure S8), which allows us to precisely locate the same molecule.

Figure 4d shows the binding kinetics in Figure 4b after we assigned all the binding and unbinding events to the single molecules (a zoom-in is shown in Figure 4f for clarity). The timestamps for binding (*t*_on_) and unbinding (*t*_off_) are linked with a line, and the length *t*_cap_ = *t*_on_ – *t*_off_ represents the capture time. Most molecules only stayed on the surface for a short time, so the *t*_on_ and *t*_off_ are almost overlapped. We only plot molecules with *t*_cap_ > 3 min in Figure 4d for clarity. By plotting the histogram of *t*_cap_, we obtained the lifetime distribution of binding events (Figure 4e), with a half-life fitted to be 1.1 min. Note that only molecules with both *t*_on_ and *t*_off_ identified are included in the histogram. Those that only have *t*_on_ but did not come off after the dissociation process are plotted separately aside the histogram (blue bar). These molecules are the same ones left at the end of dissociation in Figure 4c, which are likely attached to the surface irreversibly. Accordingly, we categorize the binding events into reversible binding and irreversible binding. We note that all signals (*R*_cap_) shown in the above sections are obtained using the irreversibly bound molecules, which contain massive nonspecific components. A reasonable way to filter out the irreversible nonspecific binding signal would be to examine the data in the reversible binding domain. To test our hypothesis, we performed a control experiment using a BSA coated surface, which only contributed to nonspecific signals (Figure 4g,i). The histogram of *t*_cap_ indeed showed a smaller binding half-time of 0.52 min (Figure 4h) compared to that of the anti-mTOR surface, indicating weaker interactions. We also measured a PEG2k coated surface, which has a similar nonspecific binding level as BSA (Figure S9). These results confirm the validity of using the reversible domain to improve detection specificity.

Not all the interactions in the reversible domain are specific. The next task is to quantify the specific component within the reversible domain. *t*_cap_ ranges from zero to several minutes, and the short *t*_cap_ is from unbound or weakly bound molecules, which is unlikely because of the strong specific binding between antibody and protein. Thus, the short *t*_cap_ should be rejected. To find out the connection between *t*_cap_ and specific binding, we first arbitrarily set a threshold to *t*_cap_ at 3 min, which separates *t*_cap_ into a weak binding region and a strong binding region (Figure 4e,h). After rejecting the weak binding having *t*_cap_ < 3 min, the remaining strong binding is plotted in Figure 4f,i (only the strong binding in the first 1000 imaged molecules is shown as an example). The anti-mTOR surface showed more bound molecules than the BSA surface, which is as expected. Next, we scanned the threshold from 1 s to 6 min and calculated the cumulative ratios of strong binding events for both anti-mTOR (*R*_Anti-mTOR_) and BSA surfaces (*R*_BSA_) (Figure 4j). The relative difference between *R*_Anti-mTOR_ and *R*_BSA_, defined by (*R*_Anti-mTOR_ – *R*_BSA_)/*R*_Anti-mTOR_, is a measure of the abundance of specific binding, which increases with the threshold and approaches ∼1 at threshold = 6 min (Figure 4k). This suggests almost all interactions are specific at high *t*_cap_. We also examined the MW of the filtered molecules. The MW distributions at different thresholds are plotted in Figure 4l. The distribution profiles for anti-mTOR and BSA surfaces are close except for the high-MW region at MW > 2000 kDa. The BSA surface has a higher level of high-MW molecules than the anti-mTOR surface, which indicates heavier molecules are more prone to nonspecific interactions. By integrating the high-MW region in Figure 4l (dashed square), we also found that the total ratio of high-MW molecules on BSA surface increases with the threshold value (Figure 4m). In contrast, the anti-mTOR surface does not have such high-MW bindings. Together, our results show the feasibility of using capture time to refine specific binding signals, and larger molecules are more likely to cause nonspecific binding in complex media (such as cell lysate).

## Discussion

### Mass Detection Accuracy Is Compromised for Unbound Molecules

In PSM, the MW is determined by the number of scattered photons by molecules within the evanescent field. Considering the exponential decay of the field, the same molecule scatters less photons when it is away from the surface. The same issue applies for a molecule that does not stay in the field for enough time. Consequently, the measured MW shows a wide distribution (Figure S2). This peak broadening effect can be mitigated by functionalizing the surface with antibodies to capture the molecule. Although this strategy has been used in our previous PSM studies for pure sample measurements,^22^ it is not applicable for measuring multiple types of proteins simultaneously in a mixture, such as cell lysate. This is simply because one cannot modify thousands of different antibodies to the surface at the same time, not to mention if they are available. Yet, the specific pulldown of one complex from lysate should report an accurate MW. The calibration curve in Figure 1c was obtained without using antibodies; thus, the standard deviation (s.d.) reflects the accuracy of mass measurement in mixture, and the coefficient of variation (CV) is found to be 38.5%. As a comparison, the CV is about 19.1% for the same measurements with antibodies.^23^

### Actual Hitting Rate, Effective Hitting Rate, and Imaging Efficiency

The collision process of an unbound molecule to the surface includes a hitting event and a leaving event, which generate a bright spot and a dark spot on PSM image, respectively (Figure 2b). If the collision takes place faster than the camera frame rate, the molecule will not be imaged because the bright spot and the dark spots “cancel out”. Therefore, for a mixture sample with various unbound molecules, the recorded images only show a portion of the hitting molecules. Next, we attempt to find a connection between them. Theoretically, the particle collision frequency or the actually hitting rate, *f*, can be calculated using *f* ≈ 4*Drc*,^33,34^ where *D* is the diffusion coefficient of protein (5 × 10^–11^ m^2^/s),^35^*r* is the radius of the imaging area (∼6 μm), and *c* is the protein concentration. The number of hitting events recorded by PSM (or effective hitting rate) is found to be proportional to the sample concentration, given by *f*_eff_ = *kc*, as determined by measuring different concentrations of IgM (Supplementary Note 3 and Figure S3). Thus, the hitting event imaging efficiency is *E* = *f*_eff_/*f* = 4.0 × 10^–5^. This number implies that the collisions of most unbound molecules are not recorded. For bound molecules, however, they do not leave the surface after hitting, so all the hitting events should be recorded.

### A Glance of the Intracellular Protein Complex Number

Using the hitting rate, we can estimate the total number of protein complexes in the cell. Figure 1h shows the hitting rate at 1 cell/10^4^ μm^2^ density is about *f*_eff_ = 1000 hits/min, which is 590 nM in concentration. Using 10^4^ μm^2^ as the surface area and 51 μm as channel height, the total number of molecules released by the single cell is 1.8 × 10^8^. Note that this number measured by PSM represents large complexes with MW > 385 kDa but not the total number of intracellular proteins. According to an estimation by Milo,^36^ the total number of proteins in a HeLa cell is about 1 × 10^10^. Thus, our result implies that the number of large complex molecules constitutes ∼2% of the total protein in HeLa cell.

### Future Design for Single-Molecule Single-Cell Detection

Understanding the heterogeneity in single cells is one of the fundamental tasks in cell biology. LFSMP has the potential for achieving single-cell resolution with single-molecule sensitivity. The bright field and the PSM can be used to locate a single cell and image the single molecules under the cell simultaneously. However, to measure a single cell accurately, the cell should be well-isolated from the neighboring ones to avoid crosstalk, which is not the case in our current flow channel design (Figure S5). A feasible solution is using microwell arrays that can trap the released molecules. An additional benefit of using microwells is that the small volume (e.g., 30 × 30 × 30 μm^3^)^37^ concentrates the molecules and increases the hitting frequency. Assuming an adherent single cell in a microwell corresponds to 60% confluence, *f*_eff_ = 10^4^ hits/min can be readily achieved (Figure 1h).

### Trade-off between Specificity and Detection Time

The real-time kinetic measurement permits excluding weak interactions and improving the binding specificity, but it takes a longer time. As shown in Figure 4j, counts decline dramatically with a higher threshold. Although the 6 min threshold for anti-mTOR can keep >90% specific interactions (Figure 4k), the remaining counts are only 0.55% of the total counts (Figure 4j). A longer sampling time is needed to collect more counts; otherwise, the data may suffer from digital counting noise. Protein complex disassembly in the lysis buffer is an additional concern for long-term detection. A balance between detection time and specificity should be reached, which needs further investigation.

## Conclusion

We have demonstrated LFSMP as a label-free intracellular protein analysis tool with single-molecule imaging capability. By functionalizing the surface with antibodies, protein complexes of interest can be specifically pulled down from the cell lysate with minimal perturbation to the native composition, allowing signaling pathway studies and real-time binding kinetics analysis. Although the current design of the LFSMP flow channel is simple, it can still measure as low as 25 cells/mm^2^. We anticipate the integration with microfabrication techniques will develop LFSMP into a powerful single-molecule single-cell analysis platform.

## Methods

### Experimental Setup

The incident light of PSM was an 80 mW laser (OBIS 660-75FP, Coherent) with the central wavelength at 660 nm. The light was first conditioned by a lens group and then focused onto the back focal plane of a 100× objective. The focused Gaussian beam from the objective was then projected to the prism surface using another group of lenses with an incident angle of 71° for SPR excitation. The reflected light from the gold film was collected by a CMOS camera (CM3-U3-13Y3MCS, FLIR) to assist finding the correct SPR angle. The scattered light from the gold film surface was collected by a 60× objective (Olympus, LUCPLFLN60X, NA = 0.7) and imaged by another CMOS camera (MQ013MG-ON, XIMEA). See Figure S1 for more details.

### Materials

Human colostrum immunoglobulin A (IgA), human plasma immunoglobulin M (IgM), and human low-density lipoproteins (LDL) were purchased from Athens Research and Technology. Rabbit Raptor antibody and rabbit phospho-Raptor antibody were purchased from Cell Signaling Technology. DyLight 488 goat anti-rabbit IgG was purchased from MyBiosource. Mouse mTOR antibody was purchased from Invitrogen. Phosphate-buffered saline (PBS) was purchased from Corning. Bovine serum albumin (BSA), human thyroglobulin (Tg), 5-aminoimidazole-4-carboxamide (AICAR), poly-l-lysine, *N*-hydroxysulfosuccinimide sodium salt (NHS), *N*-(3-(dimethylamino)propyl)-*N*′-ethylcarbodiimide hydrochloride (EDC), and *O*-(2-carboxyethyl)-*O*′-(2-mercaptoethyl)heptaethylene glycol (SH-PEG8-COOH) were purchased from Sigma-Aldrich. Methyl-PEG4-thiol (MT(PEG)_4_) was purchased from Thermo Fisher Scientific. PEG2k was obtained from Nanocs. The materials and chemicals for SDS-PAGE were purchased from Bio-Rad if not stated specifically.

### Cell Culture

HeLa cells were obtained from the American Type Culture Collection. The cells were cultured in Dulbecco’s Modified Eagle Medium (DMEM; Lonza) in a humidified incubator at 37 °C with 5% CO_2_. To active the phosphorylation of Raptor, the cells were cultured in either glucose-free DMEM for 24 h or in normal DMEM with 1 mM AICAR for 1 h before the experiment. All the DMEM was supplied with 10% fetal bovine serum (Invitrogen) and 1% penicillin and streptomycin (BioWhittaker). For PSM imaging, the cells were harvested at 75% confluence, diluted, and transferred to the surface of a preassembled, poly-l-lysine treated flow channel top piece (Figure S1).

### Cell Lysis and Western Blotting

Cells were removed from the flask using trypsin-EDTA (25-300-054, Fisher Scientific) and suspended in PBS followed by incubating in ice-cold 1× lysis buffer (9803S, Cell Signaling Technology) with protease inhibitor (A32953, Thermo Scientific) for 10 min. The lysate was sonicated for 30 s and then centrifuged for 10 min at 14 000*g* at 4 °C. The supernatant was collected, and the protein concentration was measured with BCA assay (23227, Thermo Fisher Scientific). The proteins were resolved by SDS-PAGE and transferred to a PVDF membrane for Western blotting. The detection signal was amplified by enhanced chemiluminescence (PI34580, Fisher Scientific)

### Surface Functionalization

The gold film was fabricated by coating a No. 1 cover glass with 1.5 nm Cr and then 43 nm gold using an e-beam evaporator. After rinsing with ethanol and DI water each for three times, drying with N_2_, and annealing with a H_2_ flame, the gold film was soaked in a solution containing 0.2 mM SH-PEG8-COOH and 0.2 mM MT(PEG)_4_ overnight. Then, the COOH groups were activated by incubating the film with 50 mM NHS and 200 mM EDC for 20 min. Next, 300 nM antibody in PBS was immediately added to the gold surface and incubated for 1 h to allow immobilization of the protein. Note that prior to protein immobilization, the buffer was transferred to PBS using Zeba desalting columns (Thermo Scientific) if the original buffer was not pure PBS. Ethanolamine (20 mM) was used to quench the remaining active sites for 10 min. Finally, the functionalized gold film was blocked with 0.1% BSA for 10 min. The BSA blocked chips were made by incubating the NHS/EDC activated surface with 0.1% BSA for 1 h. The PEG2k blocked chips were fabricated by incubating clean gold film in 100 μM PEG2k overnight.

### Flow Channel Assembly

The flow channel consists of three parts: an antibody-functionalized gold film on the bottom, a cover glass on the top, and a spacer in between. The top cover glass (No. 1, 18 × 18 mm) was drilled with two holes (diameter, 1 mm), which served as the inlet and the outlet. Plastic tubing (AAD04103, TYGON) was connected to the holes via a small PDMS block. The flow channel spacer was made by laser-cutting a 51 μm thick double-sided tape (9628B, 3M), which sticked the top and bottom pieces together.

### Image Processing

The image sequence was recorded using XIMEA CamTool and processed using Fiji. After recording the raw image sequence, a differential image sequence was obtained by subtracting the previous frame from each frame. The common background was removed in the differential images, and single-molecule spots were revealed. After smoothing the images with the smooth function in Fiji, the single-molecule spots were counted, and the intensity was measured using TrackMate, a plugin integrated in Fiji. The intensity was measured by selecting an Airy disc sized region, which was about 1 μm in diameter.

## Supporting Information Available

The optical setup, calibration, and mechanical noise analysis of PSM; hit rate and protein concentration; fluorescence measurement of mTORC; spatial distribution of single molecules; binding kinetics analysis for anti-mTOR, BSA, and PEG2k coated surfaces (PDF)
Supplementary Movie 1. Cell debris and organelles falling on the gold surface during lysis (AVI)
Supplementary Movie 2. Cell lysis under 50 μL/min flow (AVI)
Supplementary Movie 3. Cell lysis without flow (AVI)
Supplementary Movie 4. Native and denatured cell lysates (AVI)
Transparent Peer Review report available (PDF)

## Supplementary Material

oc2c00602_si_001.pdf

oc2c00602_si_002.avi

oc2c00602_si_003.avi

oc2c00602_si_004.avi

oc2c00602_si_005.avi

## Notes

Data Availability: The data that support the findings of this study are available from the corresponding author upon reasonable request.

## Notes

Code Availability: The codes that support the findings of this study are available from the corresponding author upon reasonable request.
