# Supplementary material for: Label-Free Single-Molecule Pulldown for the Detection of Released Cellular Protein Complexes
Source: ACS Cent Sci. 2022 Aug 18;8(9):1272–81. doi: 10.1021/acscentsci.2c00602 (PMC9523780; doi:10.1021/acscentsci.2c00602)
Supplement: Supplementary file 1 — oc2c00602_si_001.pdf [file oc2c00602_si_001.pdf]

Supporting information for

Label-free single-molecule pulldown for the detection of released cellular protein complexes

Guangzhong Ma[1], Pengfei Zhang[1], Xinyu Zhou[1,2], Zijian Wan[1,3], and Shaopeng Wang[1,2]*

[1]Biodesign Center for Biosensors and Bioelectronics, Arizona State University, Tempe, AZ 85287, USA.

[2]School of Biological and Health Systems Engineering, Arizona State University, Tempe, AZ 85287, USA.

[3]School of Electrical, Computer and Energy Engineering, Arizona State University, Tempe, AZ 85287, USA.

*Email: Shaopeng.Wang@asu.edu

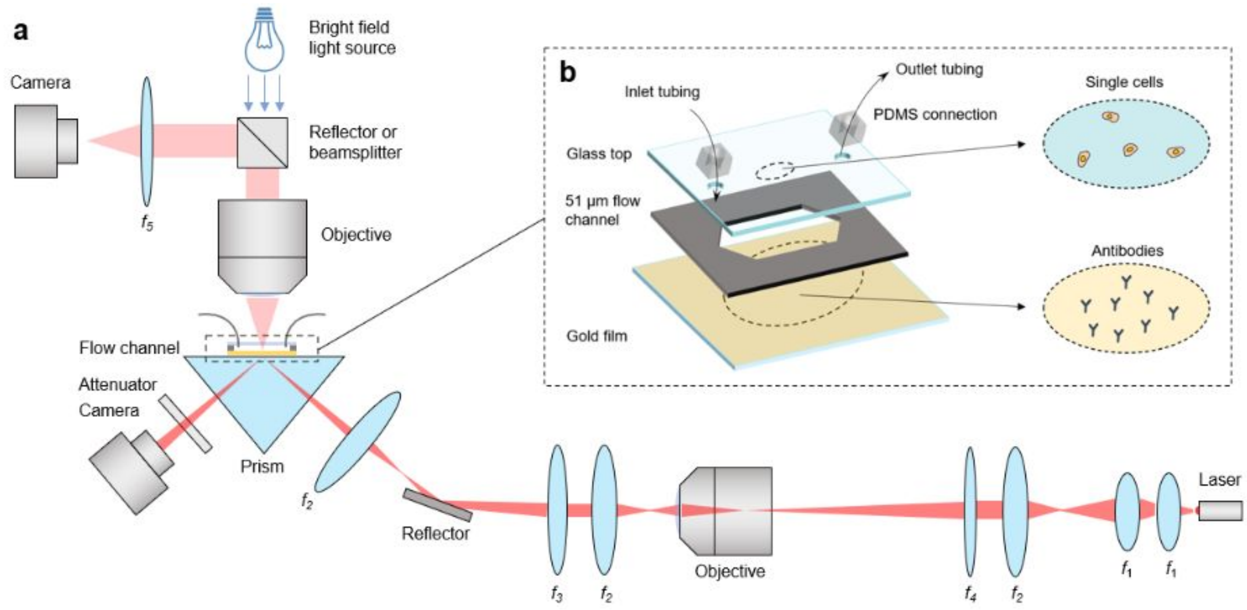

Figure S1. The configuration of PSM for single molecule detection. a, Optical configuration of prism based PSM. The laser beam is conditioned by a pair of lenses and then collimated and focused to the back focal plane of a 100× objective. The focused light from the objective is directed to the prism surface at 71° via a lens group to excite SPR. The focal length for the lenses are f_1 = 19 mm, f_2 = 30 mm, f_3 = 150 mm, f_4 = 300 mm, and f_5 = 180 mm. The reflected light from the gold film is attenuated and imaged by a camera (CM3-U3-13Y3M-CS, Point Grey), which is used for finding the correct SPR angle. The scattered light from the gold film surface is collected by a 60× objective (LUCPLFLN60X, Olympus; NA = 0.7) and imaged by a second camera (MQ013MG-ON, XIMEA). The incident light intensity is up to 5 kW/cm^2 for single molecule imaging. For bright field imaging, the reflector before the top camera is replaced with a beamsplitter. The incident light is reflected by the semitransparent gold film and imaged by the camera. **b,** Flow channel assembly. The channel is fabricated using a piece of laser-cut double-sided tape (thickness, 51 µm) as the spacer between the gold film and the cover glass. Before the assembly, the cover glass is drilled with two holes, and two small pieces of PMDS (also with holes) are bound to the cover glass for connecting the inlet and outlet tubing. Cells are cultured on the inner side of the cover glass. The gold film is modified with antibodies prior to the channel assembly. During the assembly process (~1 min), the cell and antibody surfaces should be kept wet because drying out the surfaces may reduce the cell viability and antibody activity. Solutions are delivered into the flow channel via a drug perfusion system.

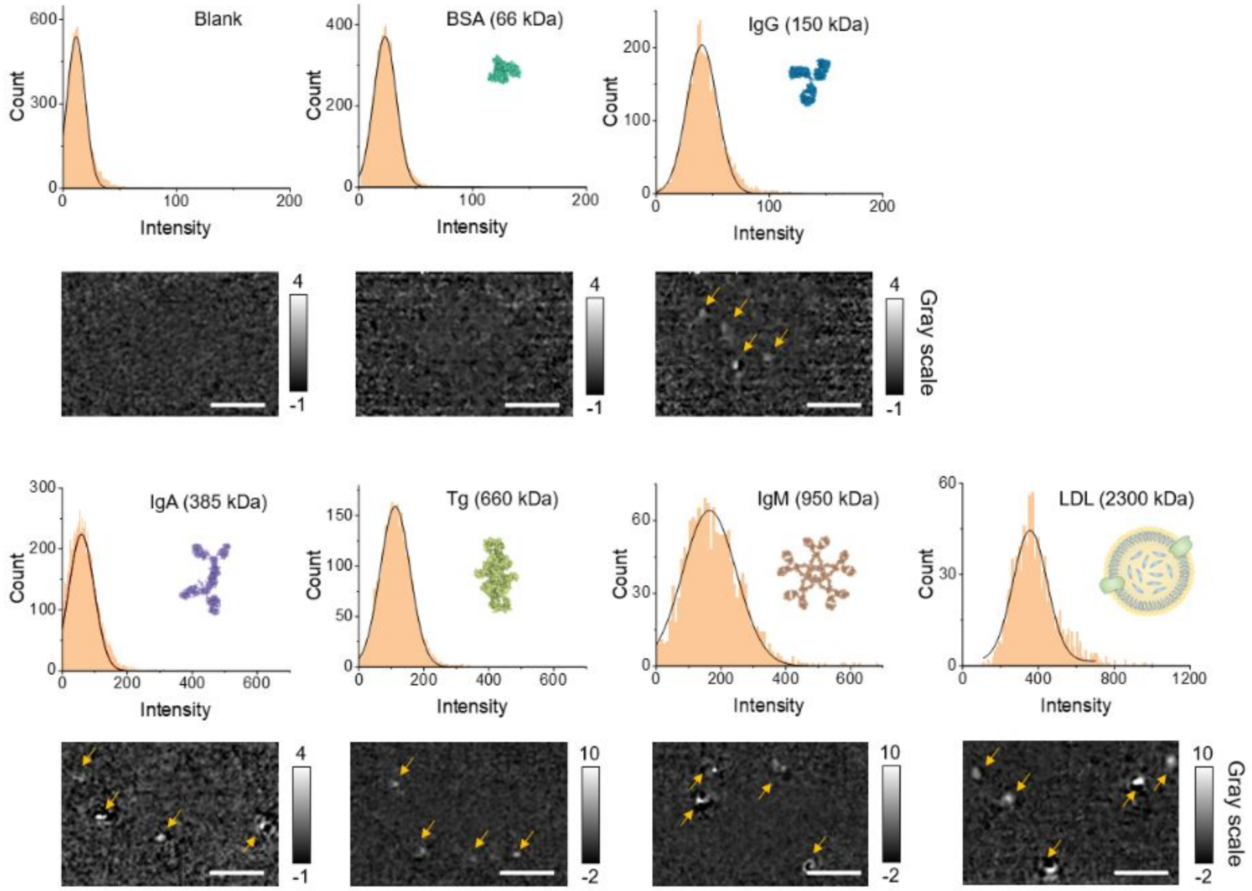

Figure S2. Single protein imaging with PSM. 6 different proteins with known molecular weight dissolved in PBS buffer and a blank (PBS buffer only) are measured with bare gold films. Representative differential images are shown in the bottom panels, with arrows pointing to the single molecules (scale bars, 5 µm). The intensities of thousands of single molecules are used to generate the histogram (top panels), which is fitted with Gaussian distribution. The power density and camera exposure time used for blank, BSA and IgG are 6 kW/cm^2 and 1 ms; and for IgA, Tg, IgM and LDL are 3 kW/cm^2 and 1 ms. All measurement results are normalized to 3 kW/cm^2 power density and 1 ms exposure time.

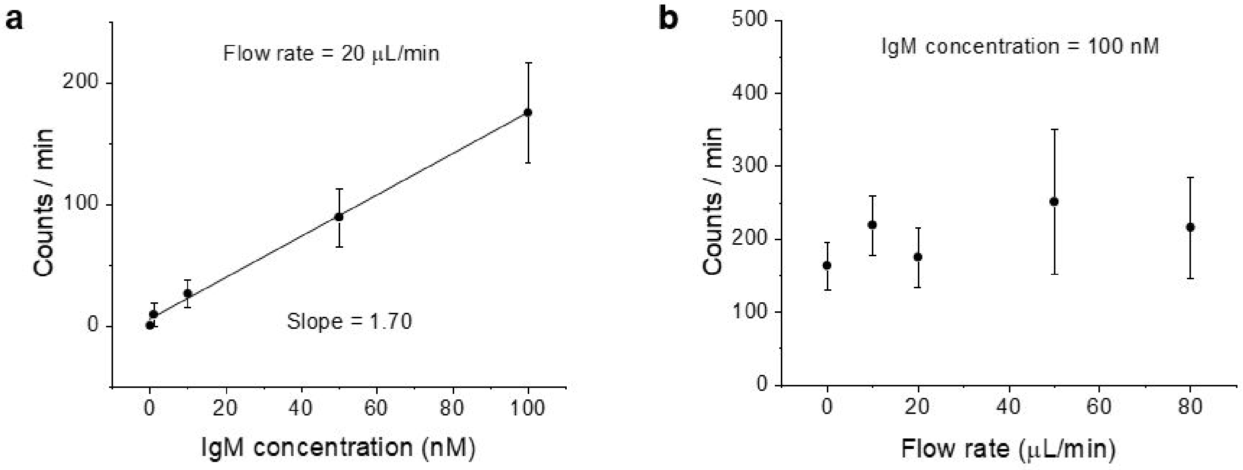

Figure S3. Effects of flow rate and analyte concentration on single molecule hitting rate. IgM collision to a BSA blocked surface was measured. **a,** Hitting rate at different IgM concentrations. The flow rate is 20 μL/min. The solid line is a linear fit of the data. The error bars represent mean ± s.d. obtained from 3 measurements (3 ROIs on the same sensor chip). **b,** Hitting rate at different flow rates. IgM concentration is 100 nM.

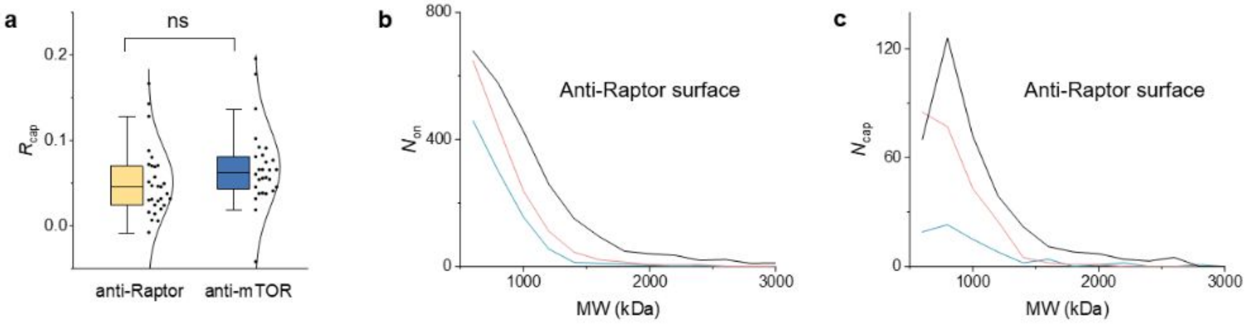

Figure S4. mTORC1 pulldown using anti-Raptor. a, Anti-Raptor and anti-mTOR has similar mTORC1 capture capabilities. The data is adapted from Figures 2f and 3d. **b,** 3 representative mass distribution curves of released molecules hitting on anti-Raptor functionalized surface. **c,** 3 representative mass distribution curves of released molecules captured by anti-Raptor.

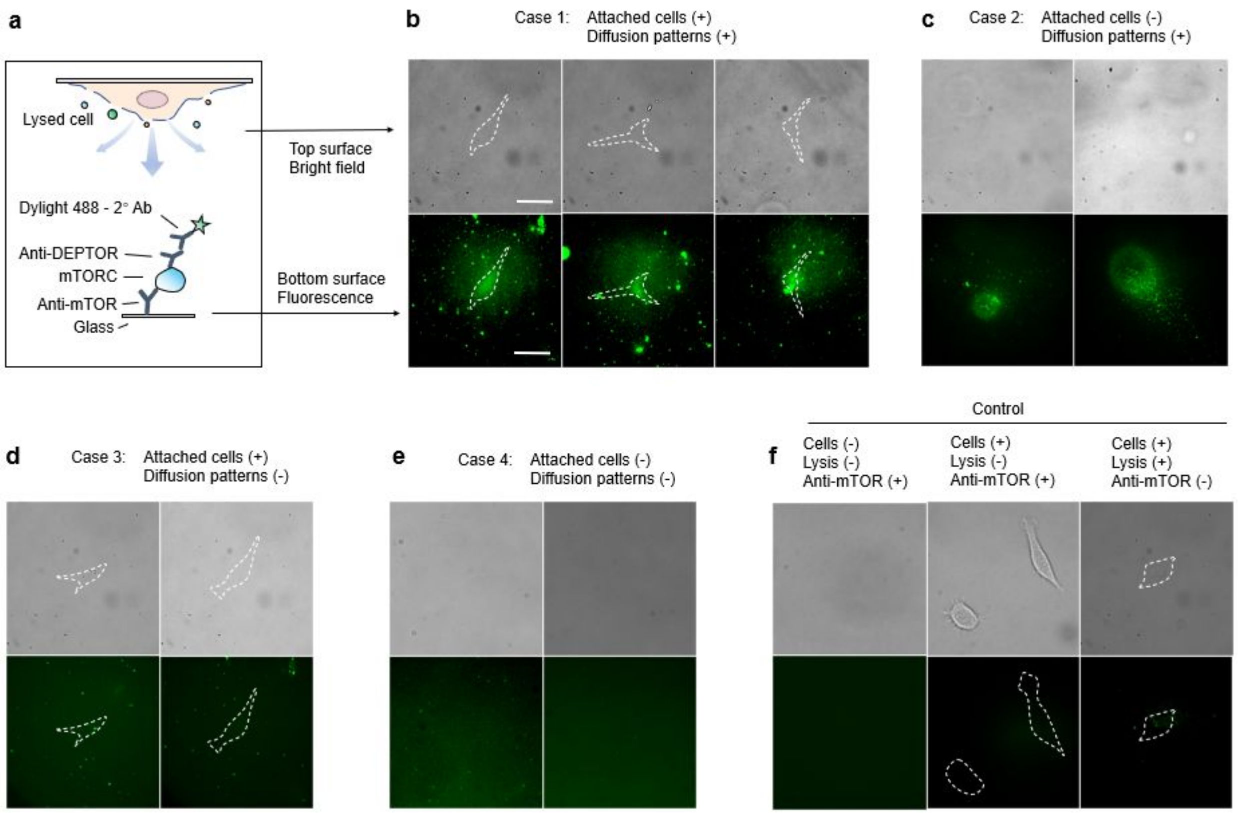

Figure S5. Fluorescence validation of cell lysis and the diffusion of released molecules. a, Detection principle. 4 different categories were found regarding the lysed cells and released molecule diffusion. **b,** Case 1: Diffusion patterns of the released mTORC were found under the cell. 3 examples are shown, and the position of the lysed cell is marked by the dashed line. Scale bar, 16 µm. **c,** Case 2: Diffusion patterns were found on the bottom surface; however, no lysed cells were found on top. **d,** Case 3: Lysed cells were observed on the top surface, but the bottom surface only has weak signals. **e,** Case 4: There were no attached cells or diffusion patterns, but the bottom surface has weak fluorescence. **f,** Control experiments showing no fluorescence signal.

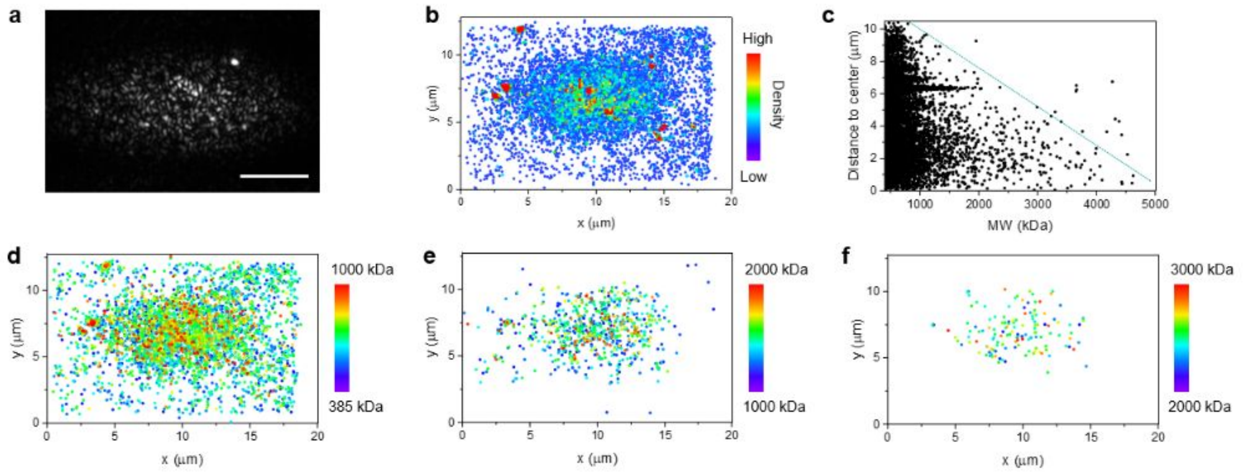

Figure S6. The spatial distribution of single molecules observed under a single cell is determined by illumination rather than diffusion. a, A raw PSM image of a BSA coated gold film surface with a single cell adherent to the top glass surface of the channel. The bright spot shows the region illuminated by the incident laser beam. Scale bar, 5 µm. **b,** The cell is lysed and the positions of single molecule hits are presented in a density map, where each data point is a single molecule. The hot spots are due to the tumbling of single molecules at nonspecific binding sites. **c,** The distance between each hit and the illumination center is plotted vs. MW of the hitting molecule. The blue line is a guide to the eye showing that molecules with higher MW are more likely to be imaged at the center of the beam. This is because molecules around the beam cannot get enough photons and are identified as smaller molecules. For this reason, only molecules imaged within the beam are counted in this work. **d-f,** Spatial distributions of released molecules with different MW.

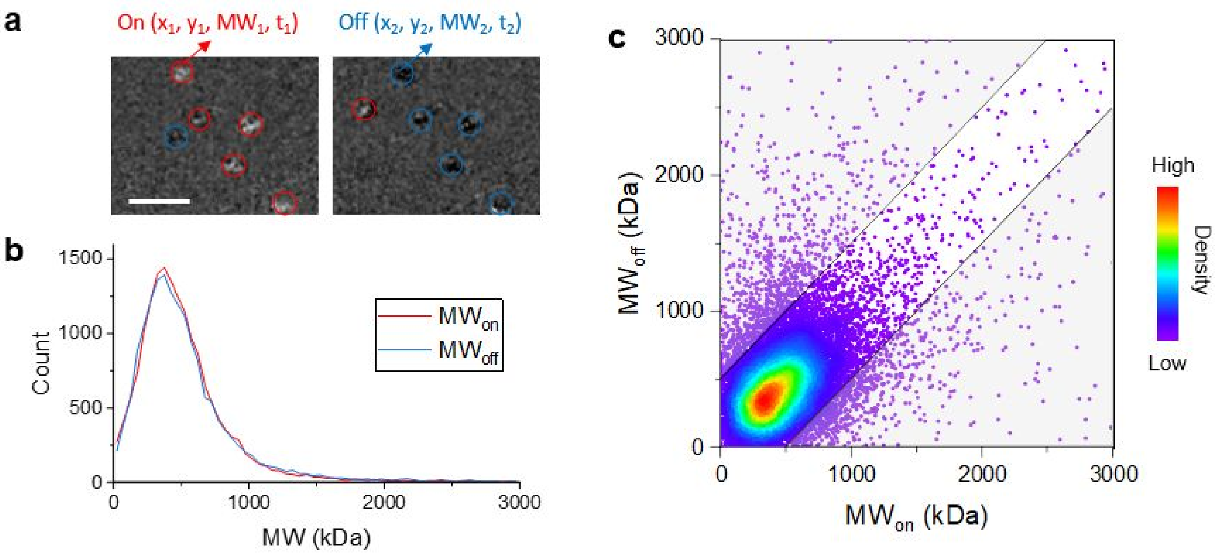

Figure S7. Correlating the binding and unbinding events of the same single molecule. a, Two consecutive frames (26 ms apart) showing the binding (on) and unbinding (off) of a few single molecules. The red and blue circles mark the on and off, respectively. For each molecule, the spatial coordinates (x,y), image intensity or molecular weight (MW), and the time stamp (t) are obtained. **b,** The MW distribution of molecules that bind or unbind the surface. **c,** Each data point is a single molecule with both identified binding and unbinding states. The MW measured during the binding and unbinding processes are plotted. Data within the shadowed regions are discarded due to large difference between MW_{on} and MW_{off}. $|MW_{on} - MW_{off}| < 500$ kDa is set as the threshold based on the measurement accuracy.

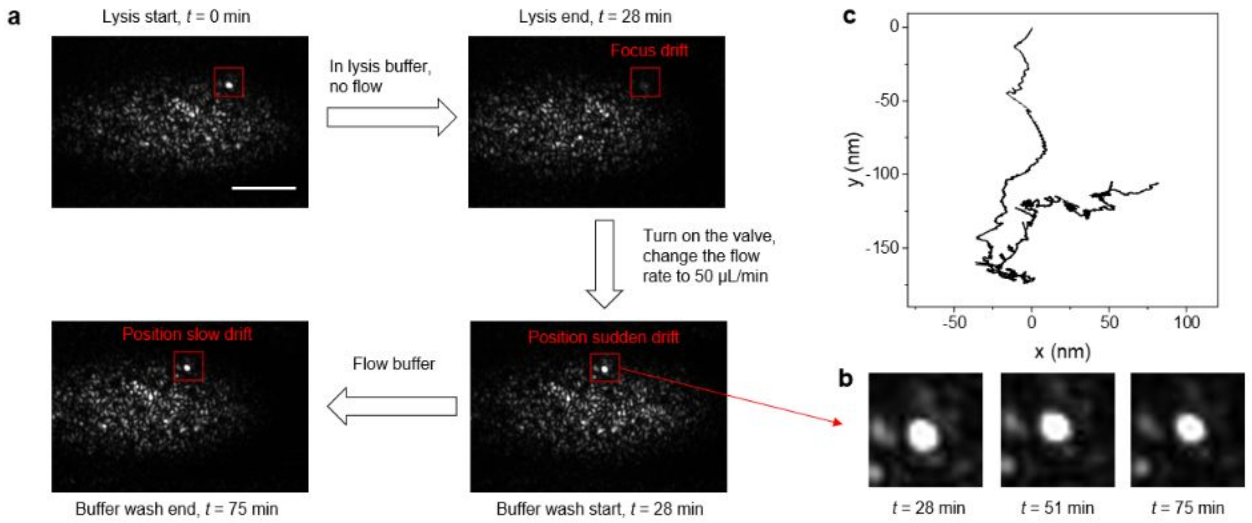

Figure S8. An example of mechanical noise and drift during the measurement. a, Raw PSM images showing a relatively large roughness on the surface as indicated by the red square, which is tracked for drift correction. During lysis buffer incubation, the focus of the roughness slightly changed, but the position remained the same. Then the valve was switched on and the flow rate was increased from 0 to 50 µL/min. A sudden position drift was observed, and at the same time, the focus resumed. The roughness continuously drifted within a small area in the next 47 min, which was tracked over time using TrackMate. Scale bar, 5 µm. **b,** Snapshots of the roughness. **c,** Tracking the drift of the roughness from t = 28 min to 75 min.

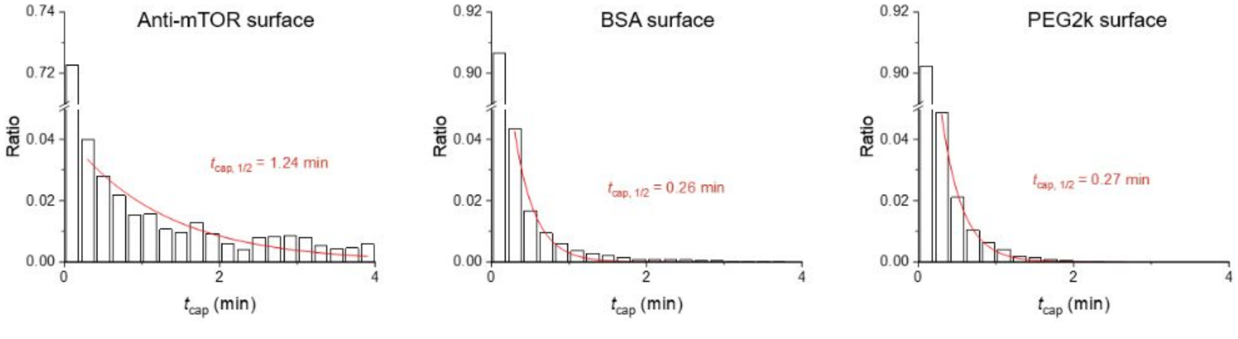

Figure S9. Additional results of cells lysed on anti-mTOR, BSA, and PEG2k surfaces, respectively. The plots show the histograms of t_{cap} and fittings of the data to exponential decay (red curves).

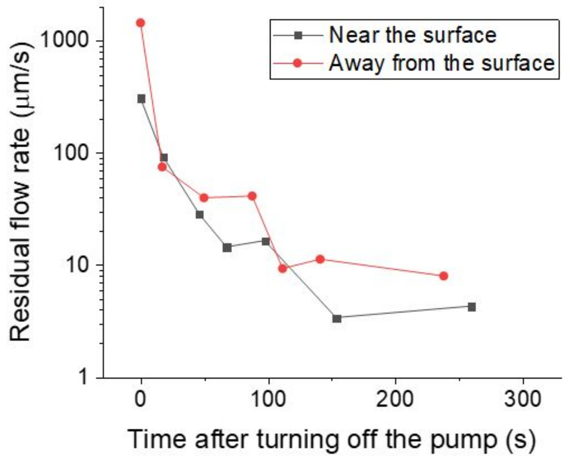

Figure S10. Residual flow rate after turning off the pump. The flow rate was measured by recording the motion of nanoparticles (polystyrene, 503 nm) in solution using the bright field mode of the PSM setup. We focused the objective at the bottom surface and away from the surface (near the channel center), respectively, to record the in-focus particles and determine the flow rate. The recording was started right after the valve was turned off.

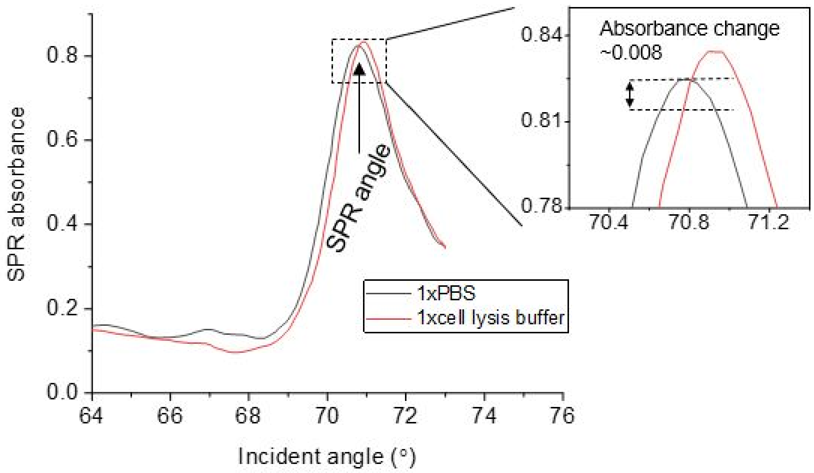

Figure S11. The effect of buffer refractive index change on scattering intensity is negligible. The plot shows the absorbance change upon switching the buffer from 1×PBS to 1×cell lysis buffer. The inset is a zoom-in of the marked region. The absorbance change at the SPR angle is 0.008.

Supplementary Note 1. Effect of lysis buffer on mTORC1 integrity

The lysis buffer we used for the pulldown experiment contained 1% Triton X-100, which was found to reduce the stability of mTORC according to previous reports.[1] Also, Jain et al have found that ~85% of mTORC are dissembled in the Triton-containing buffer in their single-molecule fluorescence measurement.[2] Yet, we expect less mTORC disassembly occurred under our experiment conditions, because the released molecules were immediately captured after cell lysis without additional treatments. This is supported by the result that the R_{cap} value obtained with anti-Raptor is only slightly lower than that obtained with anti-mTOR (Figure S4). A better and safer way to extract mTORC from cells is using buffer containing 0.3% CHAPS.[2-3] However, we found CHAPS buffer was unstable under the PSM laser illumination, possibly due to the laser induced heating effect and micelles formed by the detergent.[4-5]

Supplementary Note 2. Fluorescence detection of mTORC and diffusion after lysis

We used fluorescence to verify the release of mTORC from lysed cells. The bottom surface of the channel was a cover glass with anti-mTOR immobilized to capture the mTOR subunit of mTORC. After filling the channel with lysis buffer, the flow was immediately stopped. The cells were incubated in the lysis buffer for 20 min to allow releasing and binding of the molecules, and then the surface was washed by PBS. Then the complex was sandwiched by anti-DEPTOR (Rabbit DEPTOR antibody, MyBiosource), which recognized another component of mTORC called DEPTOR. Finally, a DyLight 488-labelled secondary antibody (2° Ab) was used to produce fluorescent signal (Figure S5a). The bright-field image of the top surface (cells) and the fluorescence image of the bottom surface (captured molecules) were captured with a fluorescence microscope (Olympus IX81).

The released mTORC shows a diffusion gradient pattern under the lysed cell (Figure S5b, case 1), which indicates the flow is stopped upon cell lysis.[6] The large and bright spots in the fluorescence image are due to the cell debris, which is also observed by PSM (Supplementary Movie 1). For some diffusion patterns, we could not find the lysed cell on the top surface, probably because the broken cell was washed off during the PBS washing process (Figure S5c, case 2). However, not all the sensor chips could observe this well-defined diffusion, because the flow control is not good enough or the cell lysis is incomplete. For example, sometimes lysed cells could be observed on the top surface, but only weak fluorescence was found on the bottom (Figure S5d, case 3). This is because the released molecules were flushed away by the residual flow, or the cells were not efficiently lysed. Moreover, we could observe fluorescence signal in some regions where there was no cell on top (Figure S5e, case 4), which is evidence that residual flow exists. To confirm the fluorescence signal is not due to nonspecific interactions, we have performed control experiments with no cells, unlysed cells, or no anti-mTOR, and the results indicate that the fluorescence is due to specific binding (Figure S5f). Although fluorescence could show diffusion patterns, we did not see such patterns with PSM because the field of view is small. The spatial distribution of single molecules observed by PSM is limited by the region of illumination (Figure S6).

We have determined the decay of flow rate after turning off the valve. It shows that the flow decreases from 500 µm/s to about 5 µm/s in 150 s (Figure S10). For cells that are lysed quickly, it is likely that the released molecules are flushed away by the flow. It seems that the diffusion patterns originate from the cells that are lysed at slow flow rate. In a nutshell, the above analysis verifies that the mTOR antibody can capture the released mTORC. It also indicates our flow system needs improvements to eliminate the residual flow. LFSMP can possibly achieve *in situ* single-cell detection with better flow control, as the single-molecule hitting rate under a cell is much higher if it is diffusion dominant.

Supplementary Note 3. Molecule concentration and hitting rate in free solution

For molecules that can be specifically captured to the surface, every hitting event can be recorded, and the hitting frequency has been found to be related to sample concentration.[7] But for molecules that do not bind to the surface or those showing "hit-and-go" behaviors, the hitting/leaving event may not be imaged. For example, if both the hitting and leaving events happen within the exposure time of the camera, they will cancel out in the image. As a result, only a portion of molecules in the mixture are imaged. To find out the relationship between hitting rate and concentration, we studied the hitting of IgM on a BSA blocked surface. IgM does not bind to BSA, so almost all the molecules are "hit-and-go". We counted the number of hitting events, plotted it vs. IgM concentration, and found a linear relationship (Figure S3a). We also measured the counts under different flow rate, and the result showed the counts were constant from 0 to 80 µL/min (Figure S3b).

Supplementary Note 4. Buffer refractive index change has negligible effect on single molecule scattering intensity in PSM

The 1×PBS (137 mM NaCl, 2.7 mM KCl, 10 mM Na_2HPO_4, and 1.8 mM KH_2PO_4) and 1×lysis buffer (20 mM Tris-HCl, 150 mM NaCl, 1 mM Na_2EDTA, 1 mM EGTA, 1% Triton, 2.5 mM sodium pyrophosphate, 1 mM beta-glycerophosphate, 1 mM Na_3VO_4, 1 µg/ml leupeptin) are different in salt concentration and hence have different refractive index. Traditional surface plasmon resonance (SPR) is known to be very sensitive to the solution refractive index as the refractive index change can shift the SPR angle, as well as the light absorbance due to plasmonic resonance if the incident light angle is fixed. In PSM, however, the absorbance is not as sensitive as SPR. This is because traditional SPR imaging set the incident angle in the high slope linear region which is lower than SPR angle to reach high refractive index sensitivity. On the other hand, PSM sets the angle exactly at the SPR resonance angle, where the sensitivity to the bulk refractive index is at the minimum, as the slope is close to zero. At the resonance angle, the absorbance or the field strength of the surface plasmon wave is the strongest, and consequently the single molecules scatter more light and achieve better signal-to-noise ratio. The sensitivity toward surface bound molecules without being interfered by the bulk solution is a unique advantage of PSM over SPR imaging.

To estimate how small the scattering intensity change is, we measured the absorbance change upon switching the buffer from 1×PBS to 1× cell lysis buffer. We scanned incident angle from 64° to 73° for 1×PBS and 1× cell lysis buffer, respectively, using a SPR microscopy (SPRm200, Biosensing Instrument). The result shows the absorbance change (or scattering intensity change) is only 0.008, which means the refractive index change has almost no effect on scattering intensity. The result is shown in Figure S11.

Supplementary Movie 1. Cell debris and organelles falling on the gold surface during lysis.

Supplementary Movie 2. Cell lysis under 50 µL/min flow.

Supplementary Movie 3. Cell lysis without flow.

Supplementary Movie 4. Native and denatured cell lysates.

Reference

1. Kim, D.-H.; Sarbassov, D. D.; Ali, S. M.; King, J. E.; Latek, R. R.; Erdjument-Bromage, H.; Tempst, P.; Sabatini, D. M., mTOR Interacts with Raptor to Form a Nutrient-Sensitive Complex that Signals to the Cell Growth Machinery. *Cell* **2002,** *110* (2), 163-175.
2. Jain, A.; Arauz, E.; Aggarwal, V.; Ikon, N.; Chen, J.; Ha, T., Stoichiometry and assembly of mTOR complexes revealed by single-molecule pulldown. *Proceedings of the National Academy of Sciences* **2014,** *111* (50), 17833-17838.
3. Chen, C.-H.; Kiyan, V.; Zhylkibayev, A. A.; Kazyken, D.; Bulgakova, O.; Page, K. E.; Bersimbaev, R. I.; Spooner, E.; Sarbassov, D. D., Autoregulation of the mechanistic target of rapamycin (mTOR) complex 2 integrity is controlled by an ATP-dependent mechanism. *Journal of Biological Chemistry* **2013,** *288* (38), 27019-27030.
4. Zhang, P.; Ma, G.; Wan, Z.; Wang, S., Quantification of single-molecule protein binding kinetics in complex media with prism-coupled plasmonic scattering imaging. *ACS sensors* **2021,** *6* (3), 1357-1366.
5. Qin, X.; Liu, M.; Yang, D.; Zhang, X., Concentration-Dependent Aggregation of CHAPS Investigated by NMR Spectroscopy. *The Journal of Physical Chemistry B* **2010,** *114* (11), 3863-3868.
6. Wang, X.; Park, S.; Zeng, L.; Jain, A.; Ha, T., Toward single-cell single-molecule pull-down. *Biophysical journal* **2018,** *115* (2), 283-288.
7. Wo, X.; Li, Z.; Jiang, Y.; Li, M.; Su, Y.-w.; Wang, W.; Tao, N., Determining the Absolute Concentration of Nanoparticles without Calibration Factor by Visualizing the Dynamic Processes of Interfacial Adsorption. *Analytical Chemistry* **2016,** *88* (4), 2380-2385.
