# Supplementary material for: Label-Free Single-Molecule Pulldown for the Detection of Released Cellular Protein Complexes
Source: ACS Cent Sci. 2022 Aug 18;8(9):1272–81. doi: 10.1021/acscentsci.2c00602 (PMC9523780; doi:10.1021/acscentsci.2c00602)
Supplement: Supplementary file 6 — oc2c00602_si_006.pdf [file oc2c00602_si_006.pdf]

oc-2022-00602v.R1

Name: Peer Review Information for "Label-free single-molecule pulldown for the detection of released cellular protein complexes"

First Round of Reviewer Comments

Reviewer: 1

Comments to the Author

Label-free single-molecule pulldown for the detection of released cellular protein complexes

S. Wang, *et al.*

oc-2022-00602v

Wang and coworkers present a label-free single-molecule pulldown (LFSMP) technique for the imaging of released cellular proteins and complexes. The authors claim that LFSMP can achieve single-molecule sensitivity at low sample loading - on the order of 25 cells/mm^2. LFSMP can directly image molecules captured on a surface using plasmonic scattering and quantify binding kinetics. The work purports to demonstrate the principle of this technique, studies the phosphorylation of protein complexes involved in a signaling pathway, and investigates kinetic analysis in pulldown specificity. While this work adequately illustrates the capabilities of LFSMP, some of the arguments surrounding the demonstration of the detection principle are not convincing. The most significant scientific issue is the evidence for the actual presence of single molecules as opposed to aggregates of several small molecules. A more convincing argument should be made for the claim that the image features of interest arise from single molecules. In this regard, addressing the questions below would go a long way toward developing this convincing argument. These issues need to be addressed before the manuscript can be considered further for publication.

1. (Page 3, lines 7, 30, and 37): How do the authors know they are imaging single molecules? Nothing is presented to definitively show the images arise from single molecules and not from aggregates, for example.

2. (Page 3, line7): Was the intensity measured over the entire image or just the bright spots?

3. (Page 3, line 10): Noise levels can vary widely from image to image even with blanks. How were these variations addressed from image-to-image throughout the experiment?

4. (Page 3, line 25): How do the authors know the extremely strong signals arise from debris and organelles? Is this a hypothesis or is there evidence to support the claim?

5. (Page 3, line 49) What is Figure 1e showing? I understand it is meant to show the cell lysis process when the channel is closed, but I do not understand what response is being measured or how it is being conveyed.

6. (Page 4, line 42): Please elaborate on the comparison of N_{cap} between the two surfaces. It is unclear how this differentiates non-specific binding events from specific binding events.

7. (Page 5, line 5): Please elaborate more why the binding can be distinguished statistically because of the wide spectrum. It is unclear what the authors mean.

Minor Issues

1. (Page 7, line 10): Figures 4d and 4g are difficult to read. Can these be cleaned so the thick band of points is not dominating the data shown?

2. Many of the figures are too small and are difficult to read. If the figures can be enlarged, it would improve their readability.

Reviewer: 2

Comments to the Author

In this paper, the authors have demonstrated a novel label-free single molecule pulldown (LFSMP) technique for imaging of released cellular protein and protein complexes with single-molecule sensitivity and low sample consumption. The reviewer strongly supports the publication of the paper in ACS Central Science due to: 1) The novelty: the proposed LFSMP platform is a novel label free technique that could be used for long-term and continuous imaging since it does not have photobleaching problem; 2) This paper represents a big step forward to the real clinical applications of using label-free imaging and sensing techniques, which could lead to a really powerful biomolecular analysis tool that could be used in the real clinical application in the near future. Currently, the label-free technique is still having limited use in clinical applications. The authors have pushed the label-free technique to the next level, and the reviewer believes this paper could be the foundation paper for the PSM using in biomolecular sensing. 3) The quality of the paper is high. The paper includes a large amount of high quality data and well-designed experiments. The data can support the conclusions very well. Therefore, the reviewer believes the paper should be published in ACS Central Science with minor revision.

1) Will the introduction of cell lysing buffer changes the refractive index compared with the running buffer? If so, that may affect the surface plasmon resonance angle and, therefore, might affect the scattering intensity. How did the authors eliminate or calibrate this effect?

2) In 2nd paragraph of page 3, the authors mentioned, “and 1000 hits/min is equivalent to 550 nM according to the calibration.” The concentration calibration figure (Figure S3) is obtained by using IgM, but the cell lysis could include all kinds of different proteins with different molecular weights. Did the authors explain the difference in the manuscript?

Author's Response to Peer Review Comments:

Dear Dr. Editor,

Please find our revised manuscript (oc-2022-00602v) entitled "Label-free single-molecule pulldown for the detection of released cellular protein complexes" submitted to ACS Central Science for publication. We have addressed all the comments by the reviewers as well as formatting issues in the revised manuscript. We respond each of the reviewers' comments below and highlight the revisions in blue in the manuscript.

Formatting Needs:

AU EMAIL: Please label as "email."

SI PG#S: The supporting information pages must be numbered consecutively, starting with page S1.

FUNDING SOURCES: Authors are required to report ALL funding sources and grant/award numbers relevant to this manuscript. Enter all sources of funding for ALL authors relevant to this manuscript in BOTH the Open Funder Registry tool in ACS Paragon Plus and in the manuscript to meet this requirement. If you have no funding to declare, please say so.

See http://pubs.acs.org/page/4authors/funder_options.html for complete instructions.

GENERAL REF FORMATTING: Periodical references should contain authors' surnames followed by initials, article title, journal abbreviation, year, volume number, and page range.

TOC MISSING: Provide a TOC image per journal guidelines (3.25 in. × 1.75 in. (8.25 cm × 4.45 cm) ; on the last page of the Manuscript) with the heading "TOC Graphic" above the graphic. Make sure to designate the file as "Graphic for Manuscript."

SYNOPSIS: The synopsis should be no more than 200 characters (including spaces) and should reasonably correlate with the TOC graphic. The synopsis is intended to explain the importance of the article to a broader readership across the sciences. Please place your synopsis in the manuscript file after the TOC graphic.

Response: We have revised the manuscript according to these requirements.

Reviewer(s)' Comments to Author:

Reviewer: 1

Recommendation: Reconsider after major revisions noted.

Comments:
Wang and coworkers present a label-free single-molecule pulldown (LFSMP) technique for the imaging of released cellular proteins and complexes. The authors claim that LFSMP can achieve single-molecule sensitivity at low sample loading - on the order of 25 cells/mm2. LFSMP can directly image molecules captured on a surface using plasmonic scattering and quantify binding kinetics. The work purports to demonstrate the principle of this technique, studies the phosphorylation of protein complexes involved in a signaling pathway, and investigates kinetic analysis in pulldown specificity. While this work adequately illustrates the capabilities of LFSMP, some of the arguments surrounding the demonstration of the detection principle are not convincing. The most significant scientific issue is the evidence for the actual presence of single molecules as opposed to aggregates of several small molecules. A more convincing argument should be made for the claim that the image features of interest arise from single molecules. In this regard, addressing the questions below would go a long way toward developing this convincing argument. These issues need to be addressed before the manuscript can be considered further for publication.

1. (Page 3, lines 7, 30, and 37): How do the authors know they are imaging single molecules? Nothing is presented to definitively show the images arise from single molecules and not from aggregates, for example.
Response: The capability of plasmonic scattering microscopy (PSM) for imaging single protein molecules has been demonstrated in our previous work (refs 22 and 23). In those works, we show how the signals are determined to be single molecules rather than aggregate by: (1) performing calibration measurements using nanoparticle of different sizes, from which we obtain a relationship between size and image intensity; (2) examining whether the detected scattering photons from single molecule matches the calculated photon number. If the signal is due to aggregates, the detected photon number would be much larger than single ones. This paper is an extension of the previous works to cellular protein detection, and it uses the same optical setup as ref 23.

For molecules released from the cells, it is possible that the molecules are not single and formed by the aggregation of smaller molecules. Even though their sizes are similar, we can still identify the molecule by using antibodies, which is the focus of this work. We have revised the manuscript accordingly (page 2, line 54) to clarify we are measuring single proteins:

"The capability of PSM for imaging single molecule has been demonstrated previously.[22,23] Here, we first measured several pure protein samples with molecular weight (MW) ranging from 66 kDa to 2.3 MDa to establish a relationship between MW and scattering intensity. The single molecules landing on the surface generated bright spots (Figure 1b, bottom panels), and the image intensity of the spots were measured."

2. (Page 3, line7): Was the intensity measured over the entire image or just the bright spots?
Response: We measured each individual bright spot. We included more details in the "Image processing" section (page 11, line 37) and on page 2 line 54:

"The intensity was measured by selecting an Airy disc sized region, which was about 1 μm in diameter."

3. (Page 3, line 10): Noise levels can vary widely from image to image even with blanks. How were these variations addressed from image-to-image throughout the experiment?

Response: The noise in the blanks is due to random fluctuations of pixel intensity, which has very low value. We set a relatively high threshold (the 385 kDa threshold shown on page 3 line 10, and the red dashed line shown in Figure 1c) to avoid picking up these noises during image processing. For the proteins with intensity above the threshold (Figure 1c), their error bars are not caused by the noises, but are likely due to the heterogeneity of individual bound molecules. The orientation of the molecule could affect the scattering intensity of the molecule.

4. (Page 3, line 25): How do the authors know the extremely strong signals arise from debris and organelles? Is this a hypothesis or is there evidence to support the claim?

Response: It is a hypothesis. Because the lysis buffer we used is mild and the released particles are immediately imaged (they do not have enough time for complete lysis), the strong signals are likely due to debris and organelles. Also, a previous fluorescence based single-molecule pull-down study (ref 14) has confirmed mitochondria or its membrane patches can be pulled down in cell lysate prepared using mild detergent. We have modified the statement on page 3 line 25 to make it clear:

"Some extremely strong scattering signals could be occasionally observed, which was likely due to cell debris and organelles (Supplementary Movie 1) because the detergent we used was mild.[14]"

5. (Page 3, line 49) What is Figure 1e showing? I understand it is meant to show the cell lysis process when the channel is closed, but I do not understand what response is being measured or how it is being conveyed.

Response: Figure 1e is a repeat of the experiment conducted in Figure 1d, the only difference is the channel is closed. It shows the released molecules are trapped inside the channel rather than being flushed away. The benefit is more molecules can be pulled down. Like Figure 1d, Figure 1e also measures the number of molecules hitting on the surface. We revised the manuscript as the following:

"We repeated the lysis experiment and measured the number of released molecules over time as we did in Figure 1d but without the flow (cells at 15% confluence), and the result is shown in Figure 1e."

6. (Page 4, line 42): Please elaborate on the comparison of Ncap between the two surfaces. It is unclear how this differentiates non-specific binding events from specific binding events.

Response: N_{cap} is the number of molecules captured on the surface. However, as we mentioned later (page 4, lines 42 to page 3, line 3), because the cell density varies between different sensor chips, N_{cap} cannot be used to differentiate specific binding from nonspecific binding events. For example, if one chip has more cells than the other, it is likely that it will have a higher N_{cap}. Therefore, we use N_{on}, which is the total number of hitting events (proportional to the cell density), to normalize N_{cap}. The normalized result, $R_{cap} = N_{cap} / N_{on}$, accurately describes the level of specific binding among different chips and differentiates specific binding from nonspecific ones.

7. (Page 5, line 5): Please elaborate more why the binding can be distinguished statistically because of the wide spectrum. It is unclear what the authors mean.

Response: We apologize for the confusion. We mean that the specific/nonspecific binding can only be differentiated by statistically analyzing the data from multiple measurements, but not from a single measurement. The reason is that the cell lysate contains many kinds of molecules, which cannot be blocked completely by the blocking reagent. Thus, some strong nonspecific binding events are expected, making it difficult to quantify the level of specific binding with one chip measurement. We have revised the manuscript to clarify this point.

"The specific and nonspecific binding of mTORC can only be distinguished statistically because the cell lysate contains many kinds of proteins and complexes, which are impossible to be blocked completely. This high nonspecific background significantly reduces the resolving power, making it difficult to differentiate specific/nonspecific binding for each individual protein binding event."

Minor Issues

1. (Page 7, line 10): Figures 4d and 4g are difficult to read. Can these be cleaned so the thick band of points is not dominating the data shown?

Response: We reduced the points in Figures 4d and 4g by plotting only those with t_{cap} > 3 min.

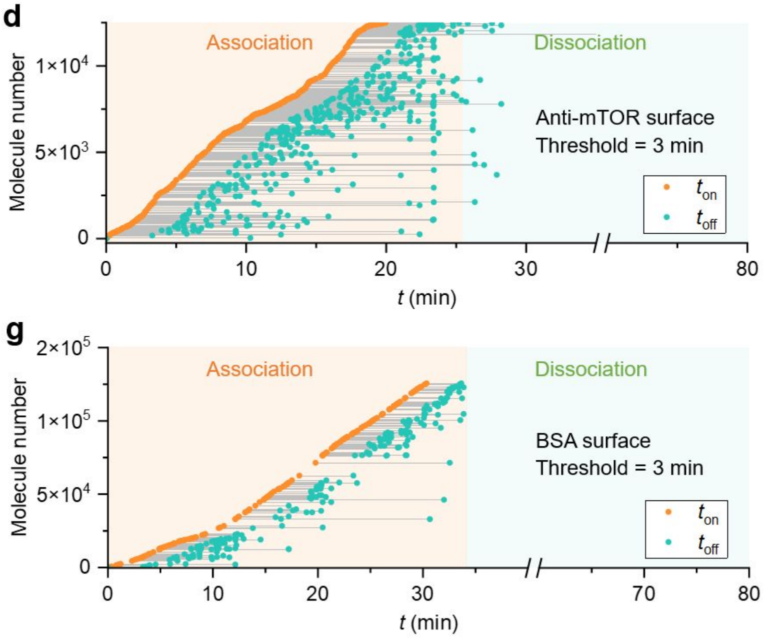

2. Many of the figures are too small and are difficult to read. If the figures can be enlarged, it would improve their readability.

Response: We have enlarged and rearranged all the figures.

Reviewer: 2

Recommendation: Publish in ACS Central Science after minor revisions noted.

Comments:
In this paper, the authors have demonstrated a novel label-free single molecule pulldown (LFSMP) technique for imaging of released cellular protein and protein complexes with single-molecule sensitivity and low sample consumption. The reviewer strongly supports the publication of the paper in ACS Central Science due to: 1) The novelty: the proposed LFSMP platform is a novel label free technique that could be used for long-term and continuous imaging since it does not have photobleaching problem; 2) This paper represents a big step forward to the real clinical applications of using label-free imaging and sensing techniques, which could lead to a really powerful biomolecular analysis tool that could be used in the real clinical application in the near future. Currently, the label-free technique is still having limited use in clinical applications. The authors have pushed the label-free technique to the next level, and the reviewer believes this paper could be the foundation paper for the PSM using in biomolecular sensing. 3) The quality of the paper is high. The paper includes a large amount of high quality data and well-designed experiments. The data can support the conclusions very well. Therefore, the reviewer believes the paper should be published in ACS Central Science with minor revision.
Response: We thank the reviewer for the positive comments. Please see our response below.

1) Will the introduction of cell lysing buffer changes the refractive index compared with the running buffer? If so, that may affect the surface plasmon resonance angle and, therefore, might affect the scattering intensity. How did the authors eliminate or calibrate this effect?
Response: We thank the reviewer for this constructive question. The lysis buffer does change the refractive index, but its effect on scattering intensity is negligible. We set the incident light angle exactly at the SPR angle, where the plasmon resonance (or light absorbance) is the strongest, to maximize the single molecule scattering signal. At this angle, however, the sensitivity of SPR absorbance to the bulk refractive index is at the minimal value. To determine how small this change is, we measured the absorbance change upon switching the buffer from 1x PBS to 1x cell lysis buffer (see below). We scanned incident angle from 64° to 73° for 1×PBS and 1× cell lysis buffer, respectively, using a SPR microscopy (SPRm200, Biosensing Instrument). The result shows the absorbance change (or scattering intensity change) is close to 0, which means the refractive index change has almost no effect on scattering intensity. We have included the above analysis and simulation result in Supplementary Note 4 and Figure S11.

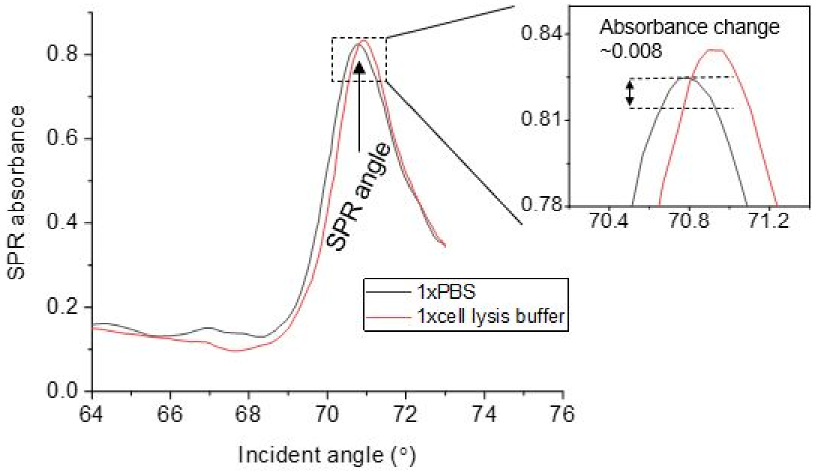

Figure S11. The effect of buffer refractive index change on scattering intensity is negligible. The plot shows the absorbance change upon switching the buffer from 1×PBS to 1×cell lysis buffer. The inset is a zoom-in of the marked region. The absorbance change at the SPR angle is 0.008.

2) In 2nd paragraph of page 3, the authors mentioned, "and 1000 hits/min is equivalent to 550 nM according to the calibration." The concentration calibration figure (Figure S3) is obtained by using IgM, but the cell lysis could include all kinds of different proteins with different molecular weights. Did the authors explain the difference in the manuscript?

Response: We count every single spot in the image that has sufficient signal-to-noise ratio (the criteria is described on page 3, line 13) to get the hitting rate. In other words, all and only the molecules that have MW>385 kDa are counted, other binding events are excluded for low SNR or cannot be resolved. In this work, we only study the molecules that are larger than 385 kDa, therefore, using IgM (950 kDa) to calibrate the concentration is appropriate. The count is related to protein concentration, not the size, as long as the protein molecule is larger than 385 kDa.
